# Large-scale discovery of male reproductive tract-specific genes through analysis of RNA-seq datasets

**DOI:** 10.1186/s12915-020-00826-z

**Published:** 2020-08-19

**Authors:** Matthew J. Robertson, Katarzyna Kent, Nathan Tharp, Kaori Nozawa, Laura Dean, Michelle Mathew, Sandra L. Grimm, Zhifeng Yu, Christine Légaré, Yoshitaka Fujihara, Masahito Ikawa, Robert Sullivan, Cristian Coarfa, Martin M. Matzuk, Thomas X. Garcia

**Affiliations:** 1 Dan L Duncan Comprehensive Cancer Center, Baylor College of Medicine, 1 Baylor Plaza, Houston, TX 77030 USA; 2 Center for Precision Environmental Health, Baylor College of Medicine, 1 Baylor Plaza, Houston, TX 77030 USA; 3 Department of Pathology and Immunology, Baylor College of Medicine, 1 Baylor Plaza, Houston, TX 77030 USA; 4 Department of Biology and Biotechnology, University of Houston-Clear Lake, 2700 Bay Area Blvd., Houston, TX 77058 USA; 5 Center for Drug Discovery, Baylor College of Medicine, 1 Baylor Plaza, Houston, TX 77030 USA; 6 Department of Molecular and Cellular Biology, Baylor College of Medicine, 1 Baylor Plaza, Houston, TX 77030 USA; 7 Department Obstetrics, Gynecology and Reproduction, Faculty Medicine, Université Laval, Quebec, QC Canada; 8 Reproduction, Mother and Youth Health Division, Centre de recherche du CHU de Québec-Université Laval, 2705 boul Laurier, Quebec, QC G1V 4G2 Canada; 9 Department of Experimental Genome Research, Research Institute for Microbial Diseases, 3-1 Yamadaoka, Suita, Osaka 565-0871 Japan; 10 Department of Bioscience and Genetics, National Cerebral and Cardiovascular Center, 6-1 Kishibeshinmachi, Suita, Osaka 564-8565 Japan

**Keywords:** Contraception, Drug target, Male reproductive tract, Paralog, Sperm maturation, Spermatid, Spermatozoa

## Abstract

**Background:**

The development of a safe, effective, reversible, non-hormonal contraceptive method for men has been an ongoing effort for the past few decades. However, despite significant progress on elucidating the function of key proteins involved in reproduction, understanding male reproductive physiology is limited by incomplete information on the genes expressed in reproductive tissues, and no contraceptive targets have so far reached clinical trials. To advance product development, further identification of novel reproductive tract-specific genes leading to potentially druggable protein targets is imperative.

**Results:**

In this study, we expand on previous single tissue, single species studies by integrating analysis of publicly available human and mouse RNA-seq datasets whose initial published purpose was not focused on identifying male reproductive tract-specific targets. We also incorporate analysis of additional newly acquired human and mouse testis and epididymis samples to increase the number of targets identified. We detected a combined total of 1178 genes for which no previous evidence of male reproductive tract-specific expression was annotated, many of which are potentially druggable targets. Through RT-PCR, we confirmed the reproductive tract-specific expression of 51 novel orthologous human and mouse genes without a reported mouse model. Of these, we ablated four epididymis-specific genes (*Spint3*, *Spint4*, *Spint5*, and *Ces5a*) and two testis-specific genes (*Pp2d1* and *Saxo1*) in individual or double knockout mice generated through the CRISPR/Cas9 system. Our results validate a functional requirement for *Spint4/5* and *Ces5a* in male mouse fertility, while demonstrating that *Spint3*, *Pp2d1*, and *Saxo1* are each individually dispensable for male mouse fertility.

**Conclusions:**

Our work provides a plethora of novel testis- and epididymis-specific genes and elucidates the functional requirement of several of these genes, which is essential towards understanding the etiology of male infertility and the development of male contraceptives.

## Background

The world human population reached nearly eight billion people in August 2019. This number continues to rise and is predicted to reach nearly ten billion by the year 2050 [1]. The increasing need to promote family planning through the development of reliable contraceptive options available to both men and women is widely recognized. Currently there are numerous contraceptive options available to women; however, identification of a safe, non-hormonal contraceptive option for men is still an ongoing challenge. Although several different fertility control alternatives for men have been investigated, none are currently clinically approved for use. Our understanding of the mechanisms underlying male reproductive physiology is still at an early stage as the identification and elucidation of the function of key reproductive proteins is still an ongoing effort. Identifying druggable protein targets expressed in the male reproductive tract has been the focus of numerous studies dedicated to the development of male contraception.

The mammalian epididymis is a segmented organ comprised of a single, highly coiled tubule with functionally and morphologically distinct regions that can be subdivided most simplistically into a proximal, central, and distal region, conventionally named the caput, corpus, and cauda regions, respectively [2]. As mammalian spermatozoa transit through the epididymis, they acquire the ability to recognize and fertilize an egg, properties that they did not possess upon exiting the testis [3]. Considering its essential role, the epididymis—in addition to maturing germ cells of the testis and spermatozoa—is a prime target for the development of a male contraceptive. To advance progress towards the development of a non-hormonal male contraceptive, several previous high-throughput studies have been published that identified a number of human, mouse, and rat genes as testis-specific or epididymis-specific [2, 4–9]. In 2003, Schultz et al. conducted the first study to identify male reproductive tract-specific genes using microarrays. Through Affymetrix-based genome-wide gene-expression analysis of meiotic- and post-meiotic spermatogenic cells, together with parallel analysis of available data from the NCBI UniGene database, the authors identified 271 mouse genes as testis-specific, which included genes with both known and unknown function at the time [4]. In the following 5 years, through two additional microarray-based studies of rat testes and purified rat testicular cells, Johnston et al. identified 58 [5] and 398 [8] additional or overlapping genes as testis-specific. In 2014, as part of the continued effort to identify novel contraceptive targets, the newer RNA-seq-based transcriptomics methodology was utilized identifying 364 human genes as testis-specific [9]. Together with antibody-based protein profiling, many of these genes were characterized in terms of the spermatogenic cell populations showing expression [9].

The first high-throughput transcriptomics study to identify epididymis-specific genes was a 2005 mouse epididymal transcriptome study, in which RNA isolated from each of the 10 epididymal segments was analyzed by microarray analysis, identifying 75 epididymis-specific genes with distinct patterns of segmental gene regulation [2]. Later in 2007, additional transcriptome profiling utilizing whole genome microarrays resulted in identification of 77 previously unreported epididymis-specific transcripts in the mouse [6] and 110 epididymis-specific transcripts in the rat [7]. A significant number of the identified mouse and rat genes in these studies were not known at the time, and only the probe identification numbers were presented.

When evaluating potential druggability in a target-based drug discovery process, one must consider the protein properties that are required for safe and effective inhibition. Among the most significant is tissue expression specificity to minimize potential adverse effects, protein function and whether protein activity or interaction with other proteins is potentially druggable, sequence similarity to closely related paralogs that may be ubiquitously expressed, and whether genetically manipulated animal models demonstrate a functional requirement for the target of interest [10]. Several noteworthy review publications have mentioned numerous genes whose critical functions, high expression, and specificity to the testes or epididymides make them viable non-hormonal male contraceptive targets [11–18]. However, among the identified genes, a significant number either (1) are required for fertility, but are expressed in non-reproductive tissues, or (2) are reproductive tract-specific, but, when disrupted, lead to subfertility [10]. In either case, both are ineffective and highly undesirable outcomes for a potential male contraceptive target. Therefore, the identification of additional novel male reproductive tract-specific genes would allow for further advances to be made in the quest to develop an effective and safe non-hormonal male contraceptive.

In this study, 21 newly acquired and 243 previously published human and mouse RNA-seq datasets [9, 19–26] were processed in parallel through a custom bioinformatics pipeline designed to identify novel reproductive tract-specific and reproductive tract-enriched transcripts. Additional databases obtained from Illuminating the Druggable Genome [27], Mouse Genome Informatics [28], and Ensembl BioMart [29] were utilized to stratify the results into subgroups based on protein druggability and on the availability of a mouse model. Numerous reproductive tract-specific and reproductive tract-enriched, potentially druggable targets for which no published mouse model exists, congruent in expression across both mouse and human datasets were identified through our analysis and verified through conventional polymerase chain reaction (PCR). We present the data in a manner that should be most relevant and of substantial interest to the male contraceptive development field since identification of new targets worthy of consideration for further functional analysis in a knockout animal model and potential drug targeting continues to be of vast importance.

Through our results, we identified four novel epididymis-specific genes (*Spint3*, *Spint4*, *Spint5*, and *Ces5a*) and two novel testis-specific genes (*Pp2d1* and *Saxo1*) worthy of functional validation in an animal model. Through the CRISPR/Cas9 system, we generated four individual gene knockouts (*Spint3*, *Ces5a*, *Pp2d1*, and *Saxo1*) and one double knockout mouse model (*Spint4/5*) revealing an essential requirement for *Spint4* and *Spint5* in male mouse fertility, and the potential utility of pursuing SPINT4 in humans as a non-hormonal contraceptive target.

## Results

### Study approaches and data

**Fig. 1:**
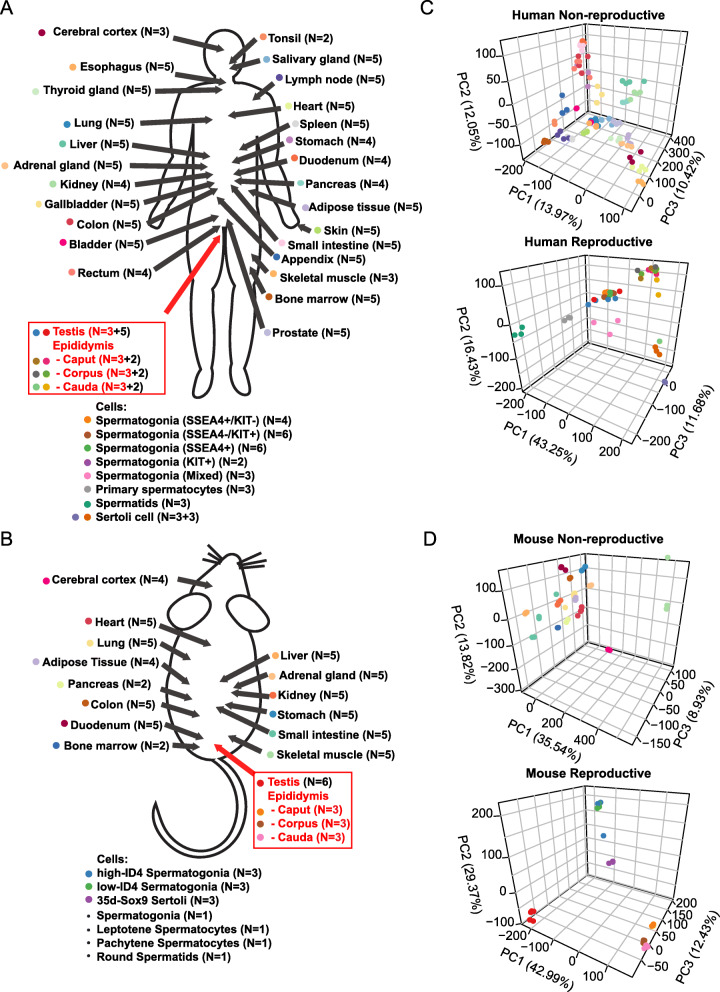
Summary of the human and mouse RNA-seq samples used in the identification of novel male reproductive tract-specific drug targets. The RNA-seq samples used in the human (**a**) and mouse (**b**) analyses are schematically shown. Principal component analysis was performed on the human (**c**) and mouse (**d**) non-reproductive and reproductive samples separately. The colors of the circles next to the tissues listed in **a** and **b** correspond to the colors used in the circles for the PCA in **c** and **d**. Sample size (*N*) values in red and/or black denote the number of new (red) and previously published (black) samples included in our analysis.

Despite significant advances in our understanding of the human and rodent testis and epididymis transcriptome, mostly through microarray-based studies, no prior studies have utilized purified human testis cells for the identification of human testis-specific transcripts, no prior studies have utilized the more state-of-the-art RNA-seq-based transcriptomics methodology for analysis of human epididymis-specific transcripts, and no prior studies have utilized RNA-seq analysis of rodent reproductive tissues or cells to identify rodent reproductive tract-specific transcripts. To address these gaps in knowledge, and to increase the number of identified reproductive tract-specific genes in both species using the most relevant high-throughput transcriptomics methodology, we analyzed in parallel on a custom bioinformatics pipeline a large number of published and newly acquired human and mouse RNA-seq datasets. One hundred and sixty-two previously published human and 81 previously published mouse RNA-seq datasets were retrieved from the Sequence Read Archive (SRA). The SRA value for each sample is listed in Additional file 1: Table S1 and Additional file 1: Table S2. We also generated 12 new human and 9 new mouse reproductive tissue RNA-seq samples (GEO Accession GSE150854). The final dataset is comprised of 3 new and 5 previously published human testis datasets [9], 27 previously published purified human germ cell datasets [23, 24], 6 previously published purified human Sertoli cell datasets [23, 26], 9 new and 6 previously published human epididymis segment datasets [21], 6 previously published mouse testis datasets [19], 9 new mouse epididymis datasets, 10 previously published purified mouse germ cell datasets [22, 25], and 3 previously published purified mouse Sertoli cell datasets [20]. An additional 118 previously published datasets contributed to the 26 non-reproductive human tissues [30] and 62 previously published datasets contributed to the 14 non-reproductive mouse tissues [19]. Figure 1a, b summarizes all the samples acquired for the study.

We performed a principal component analysis to visualize the variation in the samples after correcting for batch effects. Human reproductive and non-reproductive tissues grouped according to sample type. The reproductive tissue samples clustered by tissue type whether or not they were newly generated or acquired from the SRA (Fig. 1c). Mouse data showed a similar variation in the samples based on the tissue type (Fig. 1d). For both human and mouse reproductive tissues, samples separated by whether or not the RNA-seq was performed on isolated cells or the whole tissue. Epididymal tissue was distinct from testis tissue in both human and mouse (Fig. 1c, d).

**Fig. 2:**
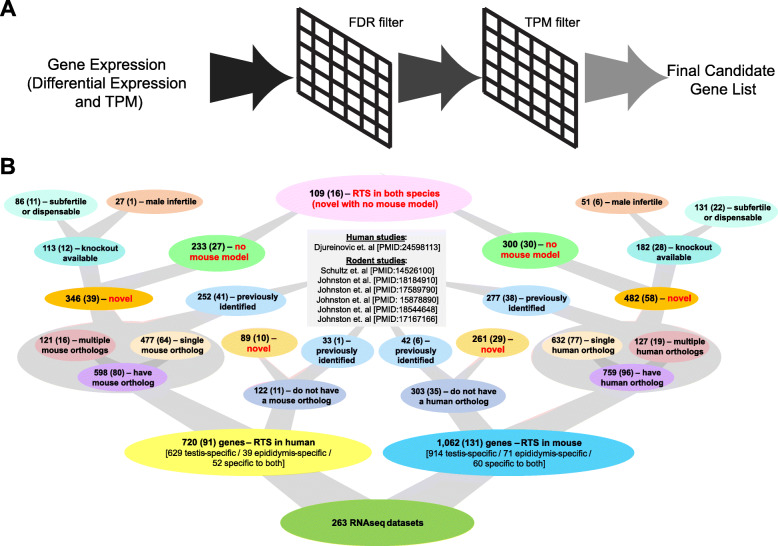
Identification of candidate drug male reproductive gene targets. **a** Diagrammatic representation of overall methodology used to identify reproductive tract-specific candidate genes in humans (720 genes) and in mice (1062 genes). The maximum gene expression was determined across all the non-reproductive tissue samples for each gene for a reproductive tissue or cell sample of interest. Genes were then filtered for significance using a false discovery rate (FDR) of less than or equal to 0.05 based on the differential gene expression analysis for the non-reproductive tissue with maximum gene expression and reproductive tissue or cell sample of interest. Genes that passed the FDR filter were filtered such that the average TPM expression value of the maximum expressing non-reproductive tissue was less than or equal to 1.0 TPM and the average TPM expression value of the reproductive tissue or cell of interest was greater than or equal to 10.0 TPM. **b** Diagrammatic representation of the number of human and mouse candidate genes in terms of (1) the number of orthologs in the opposite species, (2) the number of genes previously or not previously identified in a prior transcriptomics-based drug target report, (3) the availability and phenotypic outcome of any reported mouse models, and (4) the number of novel genes without a reported mouse model congruent across both species. The main value in each bubble represents the total number of candidate genes identified regardless of tissue or cell identified in. The numbers in parentheses comprise the total number of candidate genes that are either epididymis-specific or specific to testis and epididymis, but not testis and/or testis cell-specific only.

To identify potential male reproductive tract-specific drug candidates, we analyzed the aggregated RNA-seq data to find genes that were statistically significant in expression when compared to the non-reproductive tissue that had the maximum expression for that gene. This gene list was then further refined by filtering for genes that were lowly expressed in the non-reproductive tissue that had the maximum expression for that gene (TPM less than or equal to 1.0 for human; TPM less than or equal to 2.0 for mouse). Finally, this TPM filtered list was then filtered for the genes that had a reproductive tissue or cell expression value greater than or equal to 10.0 TPM for human, or 8.0 TPM for mouse (Fig. 2a). Across all the reproductive tissues, 720 candidate genes were identified in the human and 1304 candidate genes were identified in the mouse samples (Fig. 2b, Additional file 2: Fig. S1). Additional file 3: Table S3 and Additional file 4: Table S4 summarize the differential fold change, identity of the non-reproductive tissue with maximal gene expression based on the differential gene analysis, FDR, average and standard deviation TPM expression values, and log2 CPM gene expression value for the human and mouse samples, respectively. The results from the FDR and TPM expression value filtering for the human and mouse samples are summarized in Additional file 5: Table S5 and Additional file 6: Table S6, respectively. Additional file 5: Table S5 and Additional file 6: Table S6 report the log2 fold change for the reproductive tissue or cell of interest compared to the tissue with maximal gene expression. The genes identified in Additional file 5: Table S5 and Additional file 6: Table S6 pass the filters in at least one of the reproductive tissues or cells of interest. In Additional file 5: Table S5 and Additional file 6: Table S6, a value of zero for a given gene and fold expression comparison indicates that for that comparison, the gene did not pass the filters. The majority of genes were downregulated in the reproductive tissue of interest compared to the maximal gene expressing non-reproductive tissue (Additional file 7: Fig. S2). From the analysis, the majority of the candidate genes that passed the FDR and TPM filters were identified in the testis- or sperm-related cells in both human and mouse samples (Additional file 7: Fig. S2).

The majority of candidate genes identified in our screen that were testis-specific were already identified by the Human Protein Atlas [9] and/or our reanalysis of the HPA testis datasets (Additional file 8: Fig. S3 and Additional file 9: Table S7). Thirty-six out of the 91 genes that were identified across all the human epididymis tissue were also identified by the human combined (newly acquired and previously published datasets) testis candidate gene list. Finally, the majority of the candidate genes, 300, identified from the combined newly generated and previously published human testis datasets were shared with genes identified from the various testis cell datasets. We identified more candidate genes in the newly generated human epididymis tissues compared to previously published data: 19 out of 54 genes were unique to the newly generated caput samples compared to only 1 out of 36 genes which was unique to the previously published samples, 19 out of 75 genes were unique in the newly generated corpus samples compared to 12 out of 68 genes which were unique to the previously published corpus samples, and 33 genes were unique to the newly generated cauda samples compared to 2 genes in the previously published cauda data with no overlap between the two cauda gene lists (Additional file 8: Fig. S3 and Additional file 9: Table S7). There were 117 candidate genes that overlapped between the newly generated human testis samples and mouse testis sample gene lists, while there were 134 candidate genes that overlapped between the previously published human testis sample and mouse testis sample gene lists (Additional file 8: Fig. S3 and Additional file 9: Table S7). Across all human epididymis tissue samples, including the newly generated and previously published samples, there were 16 genes in common with the combined list of candidate genes across all the mouse epididymis tissue samples. There was a small overlap between the human and mouse samples when the newly generated human caput, corpus, and cauda tissues were individually compared to the mouse caput, corpus, and cauda tissues; there was an overlap of 10, 12, and 4 for the caput, corpus, and cauda, respectively (Additional file 8: Fig. S3 and Additional file 9: Table S7). This trend was continued for the candidate gene lists derived from the previously published human caput, corpus, and cauda samples when compared to the candidate gene list from the mouse caput, corpus, and cauda, with 7, 10, and 4 genes in common for the caput, corpus, and cauda comparisons, respectively (Additional file 8: Fig. S3 and Additional file 9: Table S7). Additional file 9: Table S7 details the genes that are unique and in common for each of the comparisons.

**Fig. 3:**
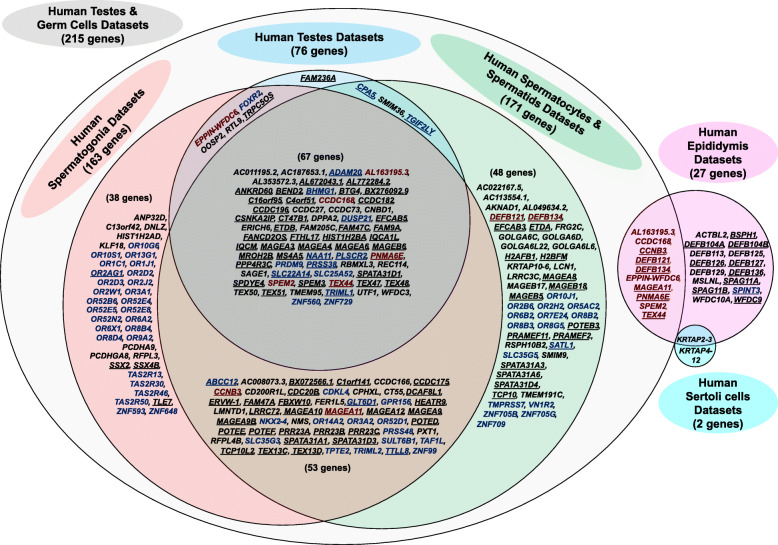
Two hundred and thirty-three novel human reproductive tract-specific genes that each have mouse orthologous genes but with no reported knockout mouse models. The listed genes were identified in one or more datasets as indicated in the Venn diagram. Underlined genes were also identified in our studies as reproductive tract-specific in mouse (109 genes). Genes written in blue encode either enzymes, kinases, GPCRs, oGPCRs, transporters, transcription factors, or proteins involved in epigenetic regulation (74 genes). Genes written in dark red were identified in both testis (testis and/or testis cell) and epididymis (10 genes).

**Fig. 4:**
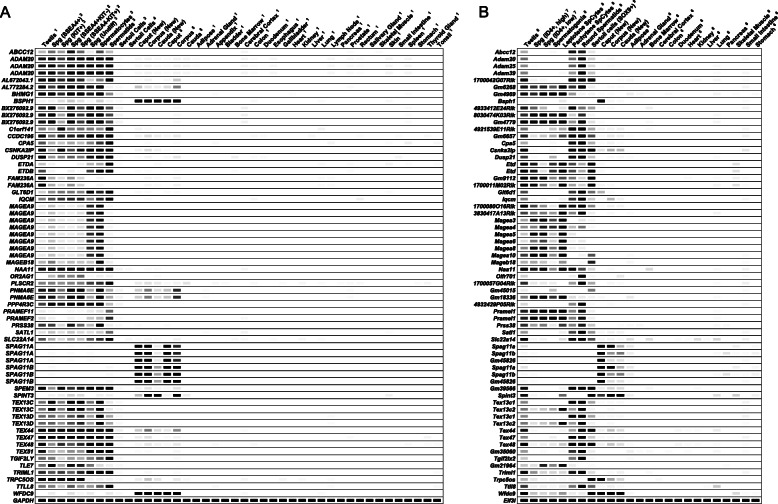
Representative novel reproductive tract-specific human (**a**) and mouse (**b**) genes without a reported mouse model. The listed genes were identified through our studies as reproductive tract-specific in both humans and mice. The digital PCR (heatmap) depicts the average transcripts per million (TPM) value per tissue per gene from the indicated human and mouse RNA-seq datasets as processed in parallel through our bioinformatics pipeline. The data was obtained from 264 published and newly acquired datasets. White = 0 TPM, Black ≥ 30 TPM. The expression profile of the human and mouse housekeeping genes, *GAPDH* and *Eif3l*, is included as reference. For data obtained from published datasets, superscript values succeeding the sample names reference the publications as follows: 1 (Djureinovic et al. [9]; Fagerberg et al. [30]), 2 (Guo et al. [24]), 3 (Zhu et al. [23]), 4 (Kumar et al. [26]), 5 (Browne et al. [21]), 6 (Consortium et al. [19]), 7 (Helsel et al. [25]), 8 (da Cruz et al. [22]), and 9 (Zimmermann et al. [20]).

**Fig. 5:**
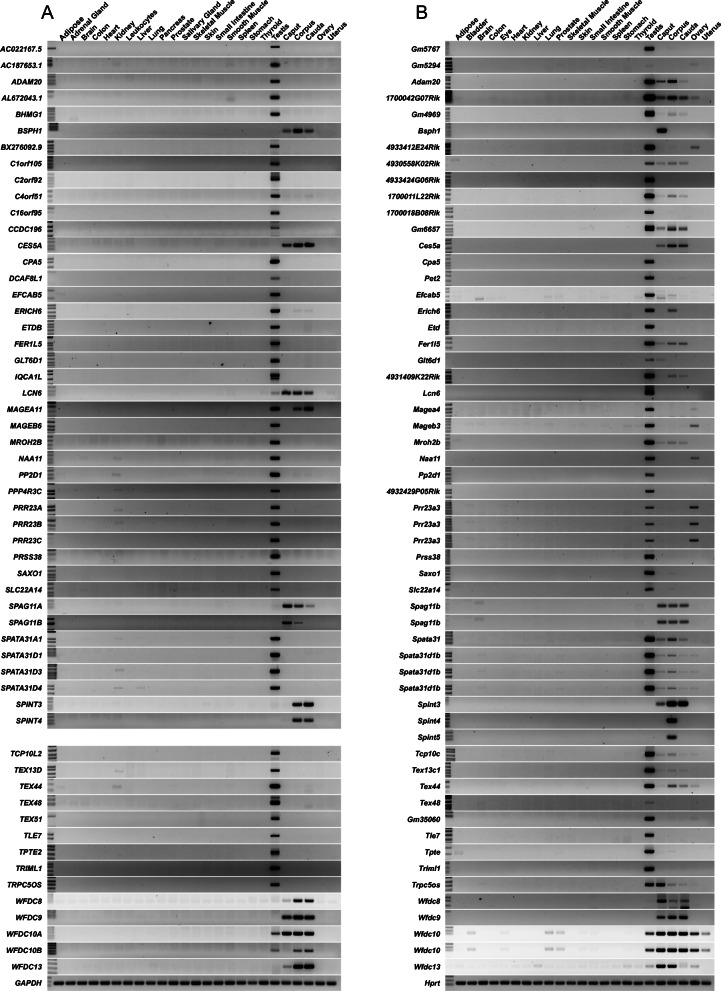
RT-PCR confirmation of reproductive tract specificity in both humans (**a**) and mice (**b**). The genes listed in this figure are novel as identified through our studies and without a reported mouse model. Humans do not have an equivalent protein-coding equivalent to mouse *Spint5*. *GAPDH* and *Hprt* are included as housekeeping genes.

To assess the potential usefulness of the candidate genes identified in each human reproductive tissue as drug targets, we assigned the genes to a protein family (i.e., GPCR or ion channel). The majority of identified genes were not from a traditional drug target family like kinases or enzymes. The testis and germ cell datasets provided the most potential targets while the epididymis datasets provided the fewest (Additional file 10: Fig. S4A). The protein family classification for each candidate gene identified in each reproductive tissue is detailed in Additional file 11: Table S8. The majority of the candidate genes do not have a reported mouse model (Additional file 10: Fig. S4B). Additional file 12: Table S9 summarizes mouse model availability for each candidate gene identified from human reproductive tissues or cells. Figure 3 shows the complete list of novel human genes without a reported mouse model as identified in each of the respective cell and/or tissue datasets. Digital PCRs (heatmap) and conventional PCRs demonstrating expression of a subset of the novel human reproductive tract-specific genes without a reported mouse model that we identified are shown in Figs. 4 and 5, respectively. Additional file 13: Fig. S5 shows the complete list of previously identified human genes that remain without a reported mouse model as identified in each of the respective cell and/or tissue datasets. Additional file 14: Fig. S6 shows the complete list of male reproductive tract-specific human genes for which a previously generated mouse model shows male infertility phenotype, as identified in each of the respective cell and/or tissue datasets.

### Reproductive tract-specific genes identified through human datasets

Through our bioinformatics analysis of previously published and newly acquired RNA-seq datasets, we identified a total of 720 genes as reproductive tract-specific in humans (Fig. 2). Of these genes, 122 genes do not have a mouse gene ortholog, while 598 genes have a mouse gene ortholog (Fig. 2). Of those with a mouse gene ortholog, 477 have a single gene ortholog (324 have the same symbol in mouse, while 153 have a different symbol in mouse), while 121 have two or more orthologous mouse genes. Seventy-six human genes had 2–3 orthologous mouse symbols, 36 genes had 4–10 orthologous mouse symbols, and 9 genes (*FAM205A*, *KRTAP10-6*, *MAGEA10*, *OR2AG1*, *PRAMEF11*, *PRAMEF2*, *SSX2*, *SSX3*, and *SSX4B*) had greater than 10 orthologous mouse symbols (11–93 symbols) (Additional file 5: Table S5). Of the 720 human genes that we identified as male reproductive tract-specific, 435 have not been previously identified in a transcriptomics-based male reproductive tract-specific study [2, 4–9]. The sum of our human data confirms the findings of 232 out of 364 genes from Djureinovic et al. [9]. After re-identification of gene symbols from reported Affymetrix IDs and consideration of orthologous genes (mouse to human and rat to human), our human data confirm the findings of 19 out of 39 genes from Johnston et al. [5], 77 out of 176 genes from Schultz et al. [4], 5 out of 32 genes from Johnston et al. [6], 36 out of 253 genes from Johnston et al. [2], 4 out of 58 genes from Johnston et al. [8], and 3 out of 19 genes from Jelinsky et al. [7]. Of the 598 genes that have a mouse gene ortholog, 346 have not been previously identified as male reproductive tract-specific, and of these, 233 human genes currently lack mouse phenotype information based on data obtained from Ensembl BioMart, MGI, IMPC, and NCBI.

#### Human testis-specific

Three hundred and eighty-six genes were identified as testis-specific through either the reanalysis of Djureinovic et al. testis datasets (377 genes identified), analysis of our de novo testis datasets (322 genes identified), or both (Additional file 5: Table S5). Three hundred and thirteen genes were congruent across both datasets, while 64 genes were uniquely identified through our reanalysis of Djureinovic et al.’s datasets and only 9 genes [*AC136352.4*, *ANKRD20A1*, *ANKRD62*, *FAM230A*, *GGTLC2*, *IQCM*, *POTEC*, *PRNT*, and *UTF1*] were uniquely identified through our de novo datasets (Additional file 5: Table S5). Interestingly, of the 377 genes we identified through Djureinovic et al.’s reanalyzed datasets, 143 were not previously identified in their report [9] or any of the other previous reports [2, 4–8]. Of these 143 genes, we randomly verified 21 of these genes as testis-specific in humans through conventional PCR (Fig. 5). We also verified through RT-PCR an additional 15 genes—such as *ALLC*, *CDKL3*, *COX7B2*, *OR2H1*, and *SPPL2C*—that had been identified through previous studies (Additional file 15: Fig. S7). Of the 386 genes identified through either testis datasets, 150 have not been previously identified; of these, 117 genes have one or more mouse orthologs; and of these, 76 genes are lacking reported phenotype information. Of the 76 novel genes lacking a reported mouse model, 7 genes encode enzymes (*ADAM20*, *CPA5*, *DUSP21*, *NAA11*, *PLSCR2*, *PRSS38*, and *TRIML1*), 6 encode transcription factors (*BHMG1*, *FOXR2*, *PRDM9*, *TGIF2LY*, *ZNF560*, *ZNF729*), 2 encode transporters (*SLC22A14*, *SLC25A52*), and 61 encode proteins of unknown drug target type (such as *ETDB*, *SMIM36*, *BEND2*, *BTG4*, *CNBD1*, *DPPA2*, *EFCAB5*, *ERICH6*, *FTHL17*, *IQCM*, *MROH2B*, *MS4A5*, *OOSP2*, *PNMA6E*, *PPP4R3C*, *RBMXL3*, *RTL9*, *SPDYE4*, *SPEM2*). All of these genes are listed in Fig. 3, and many of these genes are listed in Figs. 4, 5, and/or 6.

**Fig. 6:**
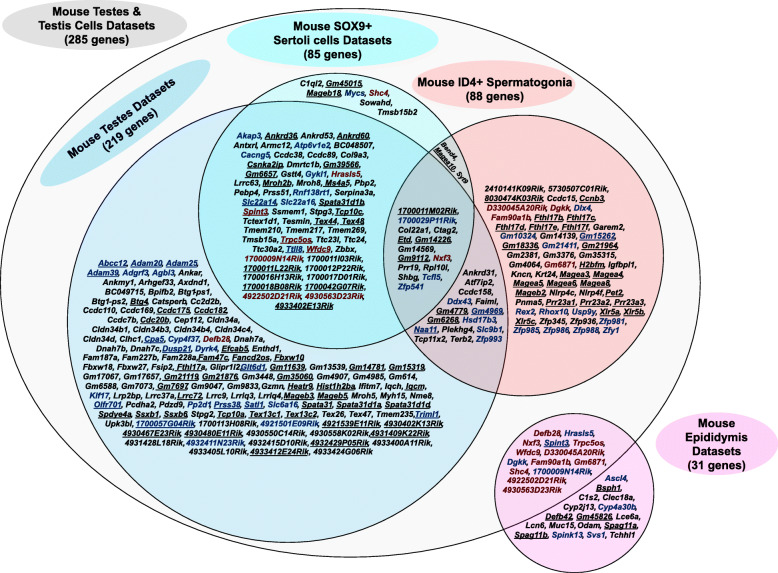
Three hundred and two novel mouse genes with human orthologs without a reported mouse model. The listed genes were identified in one or more mouse datasets as indicated in the Venn diagram. Underlined genes were also identified through our studies as reproductive tract-specific in human (111 genes). Genes written in blue encode either enzymes, kinases, GPCRs, oGPCRs, transporters, transcription factors, or proteins involved in epigenetic regulation (60 genes). Genes written in dark red were identified in both testis (testis and/or testis cell) and epididymis (14 genes).

To the best of our knowledge, no prior studies have utilized purified human testis cells for the identification of human testis-specific transcripts. Through our analysis, we identified 291 genes as human testis-specific through one or more of the human germ cell datasets, but not through either of the human testis datasets (Additional file 5: Table S5). Seventy-six genes were identified exclusively through one or more of the five human spermatogonia datasets (genes such as *ANP32D*, *C13orf42*, *DSCR4*, *OR13G1*, *OR2D2*, *OR52E4*, *SSX2*, *TLE7*), while 18 genes were identified exclusively through the human spermatocyte datasets (genes such as *H2BFM*, *MAGEB17*, *MAGEB18*, *OR2B6*, *TCP10*, and *ZNF709*) and 79 genes were identified exclusively through the human spermatid datasets (genes such as *AC013269.1*, *CLEC20A*, *OR7E24*, *PRAMEF2*, *SPATA31A3*, *TMEM191C*, *ZNF679*). Thirty-four genes were identified through all three cell types’ datasets (genes such as *CCDC166*, *ELOA2*, *FAM47A*, *HEATR9*, and *SPATA31A1*). Many of these genes are listed in Figs. 3, 4, 5, and/or 6.

Of the 291 genes identified as human testis-specific through one or more of the human germ cell datasets, 252 genes have not been previously identified, 201 of which have one or more equivalent mouse orthologs with 139 of these genes having not been knocked out in mouse. Of these 139 novel genes with no mouse model, 8 encode enzymes (*GLT6D1*, *PRSS48*, *SATL1*, *SULT6B1*, *TMPRSS7*, *TPTE2*, *TRIML2*, and *TTLL8*), 1 encodes an epigenetic protein (*TAF1L*), 6 encode GPCRs (*GPR156*, *TAS2R13*, *TAS2R30*, *TAS2R46*, *TAS2R50*, *VN1R2*), 1 encodes a kinase (*CDKL4*), 33 encode oGPCRs (such as *OR2D3*, *OR3A2*, *OR52E5*, *OR8G5*, *OR10J1*, and *OR14A2*), 6 encode transcription factors (*NKX2-4*, *ZNF99*, *ZNF648*, *ZNF705B*, *ZNF705G*, and *ZNF709*), 3 encode transporters (*ABCC12*, *SLC35G3*, *SLC35G5*), and 81 encode proteins of unknown drug target type (such as *AC008073.3*, *AC113554.1*, *AKNAD1*, *AL049634.2*, *DNLZ*, *ERVW-1*, *ETDA*, *FBXW10*, *FER1L5*, *LMNTD1*, *LRRC72*, *NMS*, *PRR23A*, *PRR23B*, *PRR23C*, *PXT1*, *RFPL4B*, and *SSX4B*). Many of these genes are listed in Figs. 3, 4, 5, and/or 6.

*FAM236A* (Figs. 3 and 4) and *OBP2B* met candidate threshold through our analysis of human testes datasets but did not meet candidate threshold from any of the germ cell or Sertoli cell datasets, indicating potential expression in peritubular myoid cells, Leydig cells, or other cell outside of the seminiferous epithelium. *FAM36A* has not been previously identified, and neither mouse orthologs (*1700011M02Rik*, *Gm9112*) have been knocked out. *OBP2B* was previously identified through Djureinovic et al. [9] and Johnston et al. [6]; however, of the equivalent mouse orthologs (*Lcn4*, *Obp2a*, *Obp2b*), only *Obp2a* has been knocked out revealing abnormal coat/hair pigmentation [31].

*ISM2* and *MAGEC2* were identified through both human Sertoli cell datasets, while also identified through testis and/or germ cell datasets. Both genes have been previously identified (*ISM2* [8], *MAGEC2* [9]). *Ism2* knockout mice display non-reproductive phenotypes [9]. Consistent with this finding, our mouse data do not identify *Ism2* as reproductive tract-specific in mice. *MAGEC2* lacks a mouse ortholog for functional analysis in mice.

#### Human Sertoli cell-specific

*KRTAP2-3*, *KRTAP4-12*, *LHX9*, and *PSG5* were identified through one or both human Sertoli cell datasets but were not identified through any of the testis or germ cell datasets indicating Sertoli cell-specific expression in the testes (Additional file 5: Table S5). None of these genes have been previously identified as reproductive tract-specific in humans although *LHX9* and *PSG5* have mouse orthologs that have been knocked out [32–37]. Human *KRTAP2-3* has mouse orthologs *Krtap5-2*, *Gm4559*, *Gm40460*, and *Gm45618*, and human *KRTAP4-12* has mouse orthologs *Krtap4-7* and *Gm11555*; none of these mouse orthologs have been knocked out (Fig. 3 and Additional file 5: Table S5).

*Psg5* knockout mice display non-reproductive phenotypes [32–36]; however, *Lhx9* knockout mice display absent testes and sterility due to an essential requirement for *Lhx9* during mouse gonad formation [37]. A *Lhx9-GFPCreER* knock-in mouse line—generated by knocking-in GFPCreER at the endogenous *Lhx9* locus—crossed with the Rosa26-tdTomato reporter mouse line revealed Cre recombinase activity in retinal amacrine cells, developing limbs, testis, hippocampal neurons, thalamic neurons, and cerebellar neurons [38]. Thus, *Lhx9* is not reproductive tract-specific in mice. Our mouse data confirm this finding.

#### Human epididymis-specific

To the best of our knowledge, no prior studies have utilized RNA-seq for analysis of human epididymis-specific transcripts. Through our studies, we identified 39 genes as human epididymis-specific through one or more of the human epididymis segment datasets that were not identified through any of the other human male reproductive tissue or cell datasets, indicating true epididymis specificity (Additional file 5: Table S5). Of these 39 genes identified as human epididymis-specific, 29 genes have not been previously identified, 24 of which have equivalent mouse orthologs with 16 of these genes having not been knocked out in mouse. Of these 16 novel human epididymis-specific genes with no mouse model, 1 encodes an enzyme-related gene (*SPINT3*) and the remaining 15 encode proteins of unknown drug target type (such as *ACTBL2*, *BSPH1*, *MSLNL*, *SPAG11A*, *SPAG11B*, *WFDC10A*, and *WFDC9*) (Fig. 3).

Seven genes were identified through our de novo sequenced human epididymis segment datasets that were not identified through our reanalysis of the human epididymis segment datasets by Browne et al.; two of these genes are considered novel without mouse models: *DEFB104A* and *DEFB104B* (Fig. 3, Additional file 5: Table S5, and Additional file 9: Table S7). Meanwhile, five genes were identified through our reanalysis of the human epididymis segment datasets by Browne et al. that were not identified through our de novo-sequenced human epididymis segment datasets; two of these genes are considered novel without mouse models: *ACTBL2* and *MSLNL* (Fig. 3, Additional file 5: Table S5, and Additional file 9: Table S7).

#### Specific to human testis and epididymis

Fifty-two genes met the criteria for identification as epididymis-specific through one or more of the human epididymis segment datasets, while also being identified as reproductive tract-specific through one or more of the testes, germ cell, and/or Sertoli cell datasets (Additional file 5: Table S5). Thus, these targets are not epididymis-specific per se, but may be desirable potential male contraceptive targets considering their broader target availability. Of these 52 genes identified as human male reproductive tract-specific and epididymis-expressed, 20 genes have not been previously identified, 15 of which have one or more equivalent mouse orthologs with 11 of these genes having not been knocked out in mouse. All 11 of these novel genes with no mouse model encode proteins of unknown drug target type (*AL163195.3*, *CCDC168*, *CCNB3*, *DEFB121*, *DEFB134*, *EPPIN-WFDC6*, *KRTAP2-3*, *MAGEA11*, *PNMA6E*, *SPEM2*, *TEX44*) (Fig. 3 and Additional file 5: Table S5).

Since model organisms other than mice may be of interest for the future functional study of human genes—especially those for which no known mouse ortholog exists—we list novel reproductive tract-specific human genes without a mouse ortholog in Additional file 16: Fig. S8, which may be of interest for generating null rat or marmoset models [39]. Digital PCR (heatmap) demonstrating expression of a subset of these novel human reproductive tract-specific genes without mouse orthologs is shown in Additional file 17: Fig. S9.

### Genes identified through mouse datasets

**Fig. 7:**
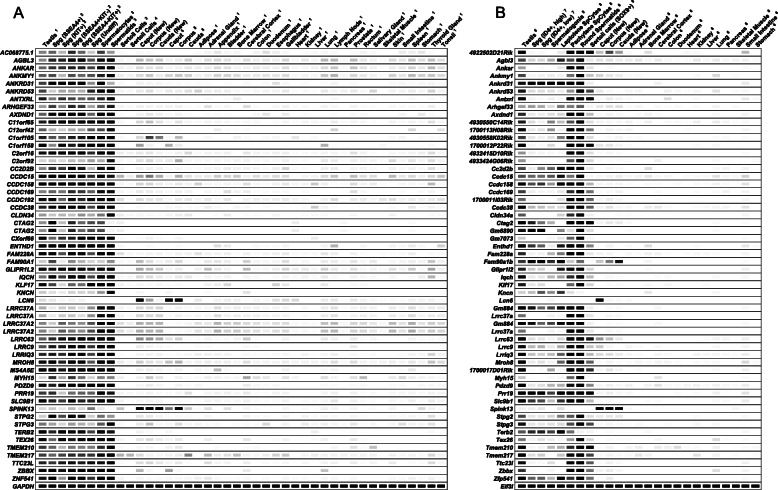
Novel reproductive tract-specific mouse genes with human reproductive tract enrichment, without a reported mouse model. The digital PCR (heatmap) depicts the average transcripts per million (TPM) value per tissue per gene from the indicated human (**a**) and mouse (**b**) RNA-seq datasets. The data was obtained from 264 published and newly acquired datasets. White = 0 TPM, Black ≥ 30 TPM. The expression profile of the human and mouse housekeeping genes, *GAPDH* and *Eif3l*, is included as reference. For data obtained from published datasets, superscript values are as previously mentioned.

Through our bioinformatics analysis of previously published and newly acquired mouse RNA-seq datasets, we identified a total of 1062 genes as reproductive tract-specific in mice. Of these genes, 303 genes do not have a human gene ortholog, while 759 genes do (Fig. 2). Of those with a human gene ortholog, 632 have a single ortholog (451 with the same gene symbol in human; 181 with a different symbol), while 127 mouse genes have two or more ortholog human genes (Fig. 2). Ninety-two mouse genes have 2–3 orthologous human symbols, 16 genes have 4–10 orthologous human symbols, and 19 genes (such as *1700080O16Rik*, *Ankrd36*, *Fam90a1b*, *Gm15319*, *Magea10*, *Pramel1*, *Spdye4a*, *Spdye4b*, and *Zfy1*) have greater than 10 orthologous human symbols ranging anywhere from twelve to twenty-six symbols (Additional file 6: Table S6). Of the 1062 mouse genes that we identified as male reproductive tract-specific in mouse, 743 have not been identified in a previous transcriptomics-based study [2, 4–9]. The sum of our mouse data confirms the findings of 150 out of 271 mouse genes from Schultz et al. [4], 7 out of 54 mouse genes from Johnston et al. [2], and 6 out of 23 mouse genes from Johnston et al. [6] (Additional file 18: Table S10). Of the 759 mouse genes that have a human ortholog equivalent, 482 have not been previously identified as male reproductive tract-specific, and of these, 302 genes currently lack mouse phenotype information based on data obtained from Ensembl BioMart, MGI, IMPC, and NCBI (Fig. 6). Digital PCR (heatmap) demonstrating expression of a subset of the novel mouse reproductive tract-specific genes with human orthologs and no reported mouse model, and with human reproductive tract enrichment, is shown in Fig. 7.

Seventeen novel genes without mouse models (*8030474K03Rik*, *Fthl17b*, *Fthl17c*, *Fthl17d*, *Fthl17e*, *Fthl17f*, *Gm15262*, *Gm18336*, *Magea3*, *Magea4*, *Magea5*, *Magea6*, *Magea8*, *Mageb2*, *Xlr5a*, *Xlr5b*, and *Xlr5c*) were identified through the mouse ID4+ spermatogonia datasets that were also identified as spermatogonia-specific through the human datasets (*BEND2*, *BX276092.9*, *FAM9A*, *FTHL17*, *MAGEA3*, *MAGEA4*, *MAGEA6*, *MAGEB6*, and *PNMA6E*) and were not identified through either mouse or human testis datasets, indicating restricted expression in spermatogonia, spermatogonial stem cells, or both (Additional file 5: Table S5 and Additional file 6: Table S6). Eight genes (*1700080O16Rik*, *Ccnb3*, *Gm21964*, *Gm4779*, *Pet2*, *Prr23a1*, *Prr23a2*, and *Prr23a3*) were identified through the mouse ID4+ spermatogonia datasets, genes whose human orthologs—*CCNB3*, *DCAF8L1*, *MAGEA10*, *MAGEA11*, *MAGEA12*, *MAGEA9*, *MAGEA9B*, *PRR23A*, *PRR23B*, *PRR23C*, and *TLE7*—were also identified through the human spermatogonia datasets. Since these genes were also identified through mouse, human, or both species’ respective testis datasets, this indicates either strong expression in the spermatogonia compartment or expression outside of and in addition to the spermatogonia compartment.

#### Mouse Sertoli cell-specific

Twenty-two genes were identified as mouse Sertoli cell-specific as they were not otherwise identified as reproductive tract-specific through our analysis of mouse testes or germ cell datasets (ENCODE Project Consortium testes and Helsel et al.’s ID4+ germ cell datasets) (Additional file 6: Table S6). Of these 22 genes, 18 have human orthologs; of these 18 genes, 17 are novel and not previously identified as male reproductive tract-specific; of these 17 genes, 7 have not been previously knocked out in the mouse (*C1ql2*, *Gm45015*, *Mageb18*, *Mycs*, *Shc4*, *Sowahd*, and *Tmsb15b2*); and of these 7 genes, 2 genes (*Gm45015* and *Mageb18*) were identified with human orthologs (*PNMA6E* and *MAGEB18*) that were also identified through our human analysis as human reproductive tract-specific (Additional file 5: Table S5 and Additional file 6: Table S6).

Unlike the limited number of reproductive tract-specific genes we identified through human Sertoli cell-specific datasets, a considerable number of genes—202 mouse genes—were identified as reproductive tract-specific through analysis of Zimmermann et al.’s mouse postnatal day 35 Sertoli cell datasets that were also identified through either the mouse testis datasets, mouse ID4+ germ cell datasets, or both (Additional file 6: Table S6). Of these 202 genes, 160 have one or more human orthologs; of these 160 genes, 54 are novel and not previously identified as male reproductive tract-specific; of these 54 genes, 36 have not been previously knocked out in the mouse; and of these 36 genes, 16 (*1700011L22Rik*, *1700011M02Rik*, *1700018B08Rik*, *1700042G07Rik*, *Ankrd36*, *Ankrd60*, *Etd*, *Gm39566*, *Gm6657*, *Gm9112*, *Magea10*, *Spata31d1b*, *Tcp10c*, *Tex44*, *Tex48*, and *Wfdc9*) were identified with human orthologs that were also identified through our analyses as human reproductive tract-specific (Fig. 6, Additional file 5: Table S5, and Additional file 6: Table S6).

#### Mouse epididymis-specific

To the best of our knowledge, published RNA-seq data of mouse whole epididymis or epididymis segments does not exist, for the identification of epididymis-specific transcripts or otherwise. Therefore, we isolated caput, corpus, and cauda segments from adult (postnatal day 60) B6/129 mice and subjected the RNA to sequencing. Sixty-six genes were identified as mouse epididymis-specific as they were not identified as mouse male reproductive tract-specific through our reanalysis of the ENCODE Project Consortium testis datasets, Helsel et al.’s ID4+ germ cell datasets, or Zimmermann et al.’s mouse postnatal day 35 Sertoli cell datasets (Additional file 6: Table S6). Of these 66 genes, 48 have human orthologs; of these 48 genes, 34 are novel and not previously identified as male reproductive tract-specific; of these 34 genes, 17 have not been previously knocked out in the mouse (*Ascl4*, *Bsph1*, *C1s2*, *Clec18a*, *Cyp2j13*, *Cyp4a30b*, *Defb42*, *Gm45826*, *Lce6a*, *Lcn6*, *Muc15*, *Odam*, *Spag11a*, *Spag11b*, *Spink13*, *Svs1*, *Tchhl1*); and of these 17 genes, 5 genes (*Bsph1*, *Defb42*, *Gm45826*, *Spag11a*, and *Spag11b*) were identified with human orthologs (*BSPH1*, *DEFB136*, *SPAG11A*, and *SPAG11B*) that are also human epididymis-specific (Fig. 6, Additional file 5: Table S5, and Additional file 6: Table S6).

#### Specific to mouse testis and epididymis

Sixty-five genes were identified as reproductive tract-specific in mouse with expression in both epididymis and testis and/or testis cell. Of these 65 genes, 48 have human orthologs; of these 48 genes, 24 are novel and not previously identified as male reproductive tract-specific; of these 24 genes, 14 have not been previously knocked out in the mouse (*1700009N14Rik*, *4922502D21Rik*, *4930563D23Rik*, *D330045A20Rik*, *Defb28*, *Dgkk*, *Fam90a1b*, *Gm6871*, *Hrasls5*, *Nxf3*, *Shc4*, *Spint3*, *Trpc5os*, *Wfdc9*); and of these 14 genes, 3 genes (*Spint3*, *Trpc5os*, and *Wfdc9*) were identified with human orthologs (*SPINT3*, *TRPC5OS*, and *WFDC9*) that are also human epididymis-specific (Fig. 6, Additional file 5: Table S5, and Additional file 6: Table S6).

### Functional validation of novel reproductive tract-specific genes

Through the aforementioned studies, we identified *Spint3*, *Spint4*, *Ces5a*, *Pp2d1*, and *Saxo1* as congruent in expression across both mouse and human datasets with expression restricted to either the epididymis (*Spint3*, *Spint4*, *Ces5a*) or the testis (*Pp2d1* and *Saxo1*) (Figs. 3, 4, 5, and 6; Additional file 5: Table S5; Additional file 6: Table S6). *Spint5* was also identified as epididymis-specific in mouse (Fig. 5, Additional file 6: Table S6); however, in humans, *SPINT5P* is a pseudogene that is not processed into protein. Conventional RT-PCR of a panel of mouse and human tissue cDNAs confirmed epididymis- or testis-restricted expression of *Spint3*, *Spint4*, *Ces5a*, *Pp2d1*, and *Saxo1* in both species and *Spint5* in mouse (Fig. 5).

To glean insight into the onset of expression for the epididymis-specific genes, *Spint3*, *Spint4*, *Spint5*, and *Ces5a*; whole epididymides from postnatal days (P) 3, P6, P10, and P14; and epididymis segments (caput, corpus, and cauda) from P21, P28, P35, and P60 aged mice were collected and analyzed through RT-PCR (Additional file 19: Fig. S10). *Spint3* expression begins as early as P3 (low) and gradually increases through P10 and P14 reaching steady levels throughout P21 to P60 in all three segments of the epididymis (Additional file 19: Fig. S10). In contrast, *Spint4* and *Spint5* display near identical expression levels with no expression at P3, P6, or P10, and expression apparent at P14 and later time points, with expression restricted to corpus only at P21 and P28, and caput and corpus, but not cauda at P35 and P60 (Additional file 19: Fig. S10). RNAscope-based fluorescence in situ hybridization revealed a distinct segment-specific pattern of expression for *Spint4* that was identical to *Spint5*, with both showing expression in most of the epithelial cells restricted to a brief region of distal caput/proximal corpus (Additional file 20: Fig. S11). *Spint3*, on the other hand, displayed a pattern of expression in a majority of epithelial cells that begins just a bit further downstream along the corpus, but persisting for much further along the corpus, throughout the corpus and into the cauda (Additional file 20: Fig. S11). These results indicate that *Spint3* shares a role that is distinct from *Spint4* and *Spint5* and indicates a potential redundancy between *Spint4* and *Spint5* and how humans may have lost the evolutionary pressure to keep *SPINT5P* as a protein-coding gene.

To glean insight into the potential spermatogenic cell population(s) expressing *Pp2d1* and *Saxo1*, we performed RT-PCR of mouse testes isolated at postnatal day (P) 3, a time point enriched for gonocytes; P6 (onset of expression of type A spermatogonia); P10 (early spermatocytes); P14 (late spermatocytes); P21 (spermatids); and P35 and P60, which display complete spermatogenesis [40] (Additional file 19: Fig. S10). Expression of *Pp2d1* and *Saxo1* is detected at similar levels at P28 and later, but not at P21 or before indicating expression during spermiogenesis and spermiation (Additional file 19: Fig. S10).

**Fig. 8:**
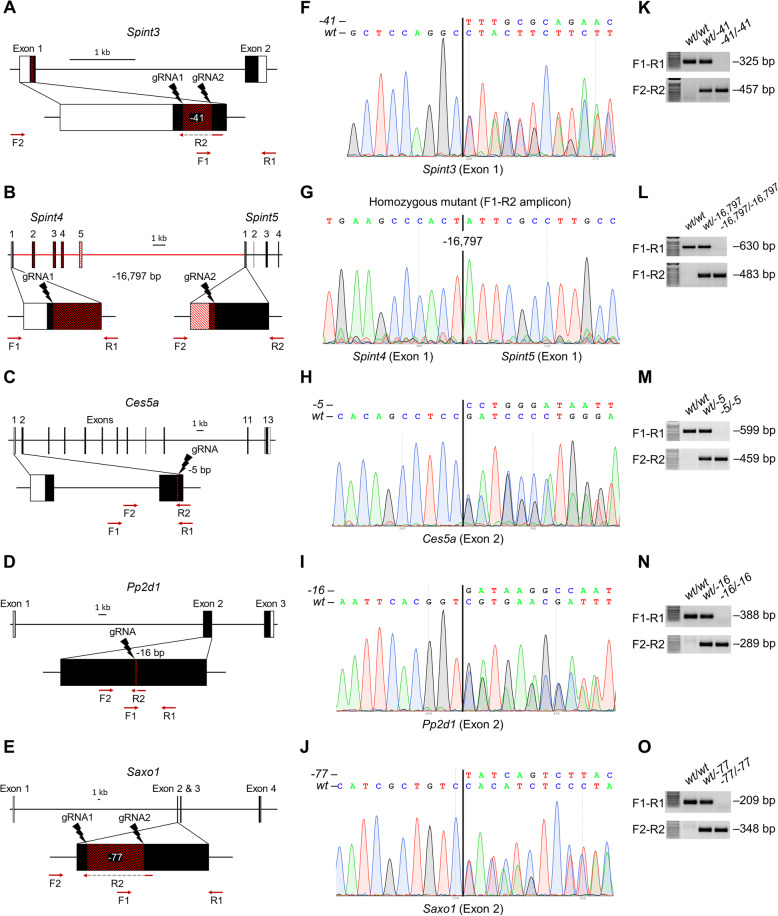
Generation of knockout mice for functional validation. **a**–**e** Genomic structure and knockout strategy for mouse *Spint3* (**a**), *Spint4/5* (**b**), *Ces5a* (**c**), *Pp2d1* (**d**), and *Saxo1* (**e**). Single or dual sgRNAs were designed to target the indicated exons in each gene. Each founder animal’s deletion mutation is indicated with red hash lines. For all but *Spint4/5*, F1 and R1 are wild-type primers and F2 and R2 are mutant primers. For *Spint4/5*, F1 of *Spint4* was combined with R2 of *Spint5* to detect the mutant allele. **f**–**j** Representative Sanger sequence result of mutant mice depicted in **a**–**e**, respectively. **k**–**o** Representative genotype result of mutant mice using the indicated primers in **a**–**e**, respectively.

To determine the male reproductive requirement and potential functional role of the identified novel male reproductive tract-specific genes, *Spint3*, *Ces5a*, *Pp2d1*, and *Saxo1* were individually ablated by CRISPR/Cas9-mediated zygote approach. Since in humans *SPINT5P* is a pseudogene, and in mice, SPINT5 protein is most similar in sequence to mouse SPINT4, we simultaneously ablated both mouse *Spint4* and *Spint5* genes, which on mouse chromosome 2 are only separated by 12.9 kilobases. The efficiency of generating each mutant is summarized in Additional file 1: Table S11. Each of the genes contained deletions of differing sizes and genomic targets. The genomic sequences flanking the deletion in each mutant are presented in Additional file 1: Table S12, and representative Sanger sequencing results for each mutant are presented in Fig. 8. Using the forward and reverse primer pairs presented in Fig. 8a–e and listed in Additional file 1: Table S13, offspring carrying the mutant alleles were identified through routine genotyping (Fig. 8k–o).

**Fig. 9:**
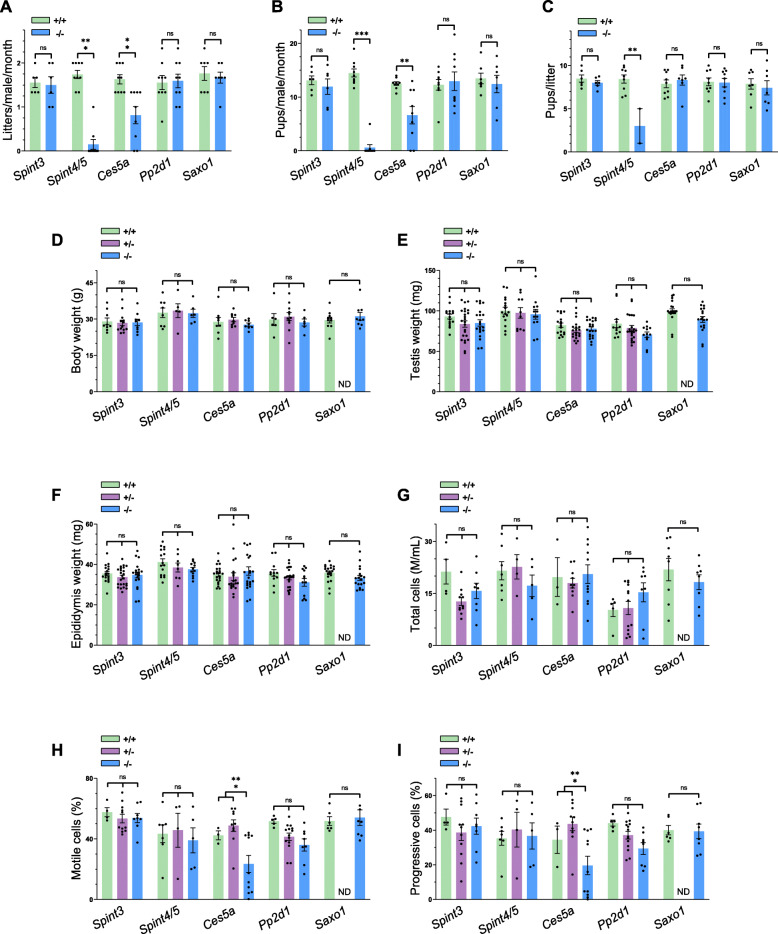
Phenotype analysis of CRISPR/Cas9 generated null mice for determining the contraceptive potential of the selected genes. *Spint4/5* and *Ces5a* null mice show significant fertility defects; meanwhile, *Spint3*, *Pp2d1*, and *Saxo1* null mice appear normal. Fertility (**a**–**c**), body and reproductive organ weights (**d**–**f**), and sperm parameters (**g**–**i**) were all measured between knockout (−/−) and littermate control [wild-type (+/+) and heterozygous (+/−)] mice as indicated. Bars represent mean ± SEM. *P < 0.05, **P < 0.01, ***P < 0.005. ns, not significant.

*Spint3*, *Spint4/5*, *Ces5a*, *Pp2d1*, and *Saxo1* knockout mouse lines were examined in parallel with littermate controls of equivalent age to determine the effect of gene ablation on spermatogenesis, sperm maturation, and fertility in male mice. None of the knockout strains generated in this study displayed any overtly abnormal appearance, difference in body weight (Fig. 9) or composition, or difference in behavior when compared to the controls.

To determine the male reproductive requirement of each of the genes of interest, *Spint3*, *Spint4*/5, *Ces5a*, *Pp2d1*, and *Saxo1* knockout and control adult male mice were housed continuously with two females for 3 months and the size and number of litters were recorded. Although *Spint3*, *Pp2d1*, and *Saxo1* knockout males sired a number and size of litters during the test mating period that was not significantly different from controls (Fig. 9a–c), *Spint4/5* and *Ces5a* knockout males sired significantly fewer litters and pups over the test mating period (Fig. 9a–c). *Spint4/5* null males displayed a statistically significant 95% reduction in the number of litters and pups sired per male and statistically significant 64% reduction in litter size, over the 3-month mating period (*N* = 9 controls, *N* = 9 KOs) (Fig. 9). Seven out of 9 males displayed complete infertility, and the two remaining males, who sired pups, sired pups at a significantly reduced number of litters and pups per month with litters of reduced litter size (Fig. 9a–c). This fertility defect in *Spint4/5* double KO males was not associated with any significant changes in epididymis and testis histology (Additional file 21: Fig. S12) or sperm numbers, motility, and morphology (Fig. 9g–i).

*Ces5a* null males displayed a variegated phenotype with an overall statistically significant 50% reduction in the number of litters and pups sired per male, but no significant difference in litter size, over a 3-month mating period (*N* = 9 controls, *N* = 9 KOs). The fertility defect in *Ces5a* KO males was associated with significant changes in epididymis histology (Additional file 22: Fig. S13) and significant reductions in sperm motility and progressive motility (Fig. 9h, i). *Ces5a* null males displayed a 50% reduction in sperm motility and progressive cells, a 50% increase in static cells, and a 25% decrease in average path velocity and progressive velocity after hyperactivation. No changes in testis histology were found (Additional file 22: Fig. S13), and despite the sperm motility defect, scanning electron microscopy failed to identify a morphological defect in *Ces5a* null sperm in comparison to controls (Additional file 23: Fig. S14).

The epididymides and testes weights of *Spint3*, *Spint4/5*, *Ces5a*, *Pp2d1*, and *Saxo1* knockout mice were not significantly different from littermate control mice (Fig. 9e, f). Histological analyses of testes from *Spint3*, *Spint4/5*, *Ces5a*, *Pp2d1*, and *Saxo1* knockout mice revealed all had seminiferous tubules with intact epithelia and the presence of all germ cell subtypes and all stages of spermatogenesis (Additional file 21: Fig. S12 and Additional file 24: Fig. S15). Histological analyses of caput, corpus, and cauda from *Spint3*, *Spint4/5*, *Pp2d1*, and *Saxo1* KO mice revealed spermatozoa in tubule lumens of all knockouts with no significant differences in epididymal histology in comparison to controls (Additional file 21: Fig. S12 and Additional file 24: Fig. S15). However, *Ces5a* knockout mice displayed significant histological abnormalities including lumen dilation (possibly from occlusion), inflammation, and the appearance of abnormal epithelia (Additional file 22: Fig. S13). Computer-assisted sperm analysis of *Spint3*, *Spint4/5*, *Pp2d1*, and *Saxo1* knockouts showed no statistically significant differences across all measured parameters including sperm concentration, sperm motility, and progressive motility (Fig. 9g–i). *Ces5a* knockouts displayed significant decreases in sperm number and sperm motility (Fig. 9h, i); however, cauda epididymal sperm isolated from a variety of *Ces5a* null animals looked morphologically indistinguishable to controls (Additional file 23: Fig. S14).

## Discussion

To date, the etiology of idiopathic male infertility is not fully understood, and hormonal male contraceptives have not been effective. Therefore, identification of novel reproductive tract-specific genes, and elucidating the functional requirement or lack thereof of these genes, is essential towards understanding the etiology of male infertility and the development of male contraceptives. Despite significant advances in our understanding of the human and rodent testis and epididymis transcriptome, mostly through microarray-based studies, no prior studies have utilized purified human testis cells for the identification of human testis-specific transcripts, no prior studies have utilized the more state-of-the-art RNA-seq-based transcriptomics methodology for analysis of human epididymis-specific transcripts, and no prior studies have utilized RNA-seq analysis of rodent reproductive tissues or cells to identify rodent reproductive tract-specific transcripts. To address these gaps in knowledge, and to increase the number of identified reproductive tract-specific genes using the most relevant high-throughput transcriptomics methodology, we analyzed in parallel on a custom bioinformatics pipeline a large number of published and newly acquired human and mouse RNA-seq datasets. Through our studies, we identified and verified many novel male reproductive tract-specific transcripts in both species, and through the CRISPR/Cas9 system, we interrogated the reproductive requirement of a subset of these genes. We found that *Spint4* (together with *Spint5*) in mice is required for normal male mouse fertility, and although not required for male fertility, we identified *Ces5a* as playing a major biological role in the epididymis. We report the remaining genes that we knocked out—*Spint3*, *Pp2d1*, and *Saxo1*—as dispensable for male reproductive function, which is essential information to disseminate to the scientific community. Our study also verified the male reproductive tract-specific expression of many previously identified genes (Additional file 13: Fig. S5, Additional file 15: Fig. S7), and genes for which previously published mouse models display male infertility phenotypes (Additional file 14: Fig. S6). This later group of already functionally validated genes serves as potential male contraceptive targets worth underscoring to the research community.

Prior to massively parallel microarray-based and RNA-seq-based transcriptomics analyses for the identification of reproductive tract-specific genes, the NCBI UniGene database was a valuable resource for many in the male reproductive biology field for identifying testis-specific transcripts [4, 41–43]. Although in our study we only considered prior microarray-based and RNA-seq-based studies when considering the novelty of the genes that we identified, it is worth noting that several genes that we identified—not previously identified in microarray-based and RNA-seq-based transcriptomics studies—were previously identified through studies that solely utilized the UniGene database [41, 42]. Nineteen human genes that we identified—*C16orf82*, *CCDC27*, *CPA5*, *FAM217A*, *FAM46D*, *FAM47C*, *FBXO24*, *FBXW10*, *FKBP6*, *GALNTL5*, *KCNU1*, *MAGEB3*, *MROH2B*, *NUTM1*, *PRDM9*, *RBMXL3*, *SPATA31E1*, *TRIML1*, and *TRPC5OS*—were previously identified by Liu et al. [42], and seven mouse genes that we identified—*1700013D24Rik*, *Akap3*, *Ankrd36*, *Hrasls5*, *Spesp1*, *Tex22*, and *Ubqlnl*—were previously identified by Choi et al. [41] and Liu et al. [42]. Thus, our results confirm the findings of these previous studies.

Since more than half of all human protein-coding genes are categorized as unknown in terms of drug target potential (Additional file 11 - Table S8), and only 30% encode for classically druggable enzymes, GPCRs, ion channels, nuclear receptors, and transporters (Additional file 10 - Fig. S4), the potential to find new undiscovered drug targets that can be drugged using classical approaches is somewhat limited. Indeed, in our study, we found one hundred and nine genes to be novel in terms of previously published high-throughput transcriptomics studies, without a current reported mouse model, and reproductive tract-specific in both humans and mice (Figs. 2, 3, and 6). Many of these genes (93 genes; 85%) fall into the category of unknown and may otherwise be considered “undruggable” due to various challenges with existing targeting approaches [10]. However, the contraceptive potential of these genes should not be overseen, but rather investigated for potential identification of a high-affinity small molecule that can either interfere with protein-protein interaction (PPI) or target the protein specifically for degradation using a new technology called Proteolysis Targeting Chimeras (PROTACs). Protein-protein interaction targets are not deemed undruggable, based on the discovery of small molecules capable of deeper and higher affinity binding within the contact surfaces of the target protein [44]. Additionally, once a high-affinity small molecule against a specific target protein is identified, an engineered PROTAC molecule can mark a target protein for proteasomal degradation by linking the target protein to the polypeptide co-factor, ubiquitin [45–47]. There are currently various combinations of PROTACs developed to overcome the limitations of cell permeability, stability, solubility, selectivity, and tissue distribution [48–51]. Therefore, disrupting PPIs or utilizing PROTACs provides the potential to greatly promote the development of contraceptive drugs against the “undruggable” non-enzymatic target protein space.

### Drug target specificity of novel targets

If gene knockouts for closely related and ubiquitously expressed paralogs display no abnormal phenotype, then unintended drug targeting of these proteins may result in no side effects in humans. However, the burden of safety for a male contraceptive is extremely high, and if it can be avoided, targeting non-reproductive tract-expressed proteins in humans should be avoided since the functional requirements for these proteins may not be fully understood. Further, although mice are one of the best models for human disease, they are unable to communicate when they are unwell, a phenomenon that may occur independent of any measurable phenotypic traits. Thus, potential reported side effects in humans during clinical trials may have been present, but missed, during animal studies, or in fact be present in humans and not in mice because of the vast biological differences across these two species. Thus, with drug safety in mind, reproductive tract-specific candidates should be prioritized based on somatic cell-expressed protein sequence similarity, especially in the drug binding pocket.

According to Ensembl, several novel reproductive tract-specific genes without mouse models that we identified and verified—*AC022167.5*, *AL672043.1*, *BHMG1*, *C1orf105*, *C2orf92*, *C4orf51*, *CCDC196*, *SPINT3*, *TEX44*, *TEX48*, *TEX51*, and *TRPC5OS* (Figs. 3, 4, and 5; Additional file 5: Table S5)—have no known associated paralogs, indicating reproductive tract-specific drug targeting is highly likely. Additionally, *SPAG11A* and *SPAG11B* are epididymis-specific paralogs (Figs. 3, 4, and 5; Additional file 5: Table S5) with no other known paralogs according to Ensembl. *EFCAB5* (Figs. 3 and 5, Additional file 5: Table S5) has a ubiquitously expressed paralog, *NSRP1*, with only 7% amino acid sequence similarity, indicating specific drug targeting potential for this candidate. Likewise, *ERICH6* and *MROH2B* (Figs. 3 and 5, Additional file 5: Table S5) have ubiquitously expressed paralogs *ERICH6B* and *MROH2A*, respectively, with 20% and 30% amino acid sequence similarity, indicating reasonable potential for specific drug targeting.

*PRR23A*, *PRR23B*, and *PRR23C* are testis-specific paralogs (Figs. 3 and 5, Additional file 5: Table S5) with *PRR23D2* as the next closest paralog according to Ensembl. Since PRR23D2 has less than 26% amino acid sequence similarity to PRR23A, PRR23B, and PRR23C, but appears to be epididymis-specific according to the Human Protein Atlas [52], all four proteins of unknown function make suitable drug candidates. Likewise, *SPATA31D1*, *SPATA31D3*, and *SPATA31D4* are testis-specific paralogs (Figs. 3 and 5, Additional file 5: Table S5) with *SPATA31A5* as the next closest paralog according to Ensembl. Since SPATA31A5 has less than 26% sequence similarity to SPATA31D1, SPATA31D3, and SPATA31D4, but also appears to be reproductive tract-specific according to HPA [52], all four proteins with unknown function also appear to be worthy of consideration for potential drug targeting. TPTE and TPTE2 are testis-specific paralogs (Figs. 3 and 5, Additional file 5: Table S5) with ubiquitously expressed PTEN as the next closest paralog according to Ensembl. Since PTEN has less than 25% sequence similarity to TPTE and TPTE2, off-target effects appear to be unlikely. Likewise, WFDC10A, WFDC10B, and WFDC13 are all epididymis-specific paralogs with the closest non-reproductive tract-expressed paralog, WFDC5, having less than 26% sequence similarity to any of the RTS paralogs, also indicating high drug specificity potential.

Several additional novel reproductive tract-specific enzyme and GPCR genes without mouse models that we identified—*GPR156*, *PRSS38*, *PRSS48*, *SULT6B1*, *TMPRSS7*, *TRIML2*, and *TTLL8* (Fig. 3, 4, and 5; Additional file 5: Table S5)—have non-reproductive tract-expressed paralogs with less than 35% sequence similarity indicating good drug specificity potential. A novel testis-specific transporter gene without a reported mouse model that we identified, *SLC25A52*, would make a poor drug candidate since its closest paralog, *SLC25A51*, is 93% similar in amino acid sequence and ubiquitously expressed. *CPA5*, *IQCA1L*, and *PPP4R3C* have ubiquitously expressed paralogs, *CPA1*, *IQCA1*, and *PPP4R3B*, respectively, with 50–60% protein sequence similarity indicating careful consideration must be made for potential drug targeting without off-target effects.

Of the seventy-three genes that our study identifies as reproductive tract-specific in humans and for which a published mouse model shows male infertility phenotype (Additional file 14: Fig. S6) [28, 29, 31], it is worth noting that 21 genes—*CNBD2*, *DEFB110*, *FAM170A*, *FBXO47*, *MEIG1*, *MEIOB*, *MEIOC*, *ODF1*, *ODF4*, *REC114*, *RNF17*, *SPACA1*, *SPATA22*, *SPEM1*, *SPO11*, *SYCP1*, *TERB1*, *TEX19*, *TEX38*, *TNP2*, and *TOPAZ*—do not have any associated paralogs and, thereby, may be considered most suitable for further drug development. However, it is also worth noting that it may be possible that genes required for male fertility in mice may not necessarily be required for male fertility in humans. Of the seventy-three human reproductive tract-specific genes our study identified with male mouse infertility phenotypes, twenty-seven genes—*ACTL7B* [53], *AKAP4* [54], *BOLL* [55], *BRDT* [55–60], *CATSPER4* [54], *CCDC155* [61], *FKBP6* [55, 61–63], *MEIG1* [64], *MEIOB* [55–57, 65], *NANOS2* [55, 61], *ODF1* [55], *PRDM9* [61, 66, 67], *PRSS37* [55], *RAD21L1* [68], *RBMXL2* [55, 62], *RNF17* [69], *SOHLH2* [61, 70], *SPACA1* [55], *SPATA16* [55, 56], *SPEM1* [55], *SPO11* [55, 58, 61], *SUN5* [55–57], *SYCP1* [55], *TEX38* [55], *TNP2* [55], *TSSK1B* [62], and *ZPBP* [55]—are currently associated with mutations underlying human male infertility, confirming a similar functional requirement for these genes in humans may exist. For the remaining 45 genes, however, either these genes are not required for human male fertility as they are required in mice, or associated mutations in male infertile patients have not yet been reported.

Although many reproductive tract-specific genes have been studied through functional genetics approaches, many remain to be solved. Elucidating the function of these novel genes is necessary to build a better understanding of the factors underlying spermatogenesis and sperm maturation, which has implications in understanding the etiology of male infertility and the development of male contraceptives. In this study, four epididymis-specific genes (*Spint3*, *Spint4*, *Spint5*, and *Ces5a*) and two testis-specific genes (*Pp2d1* and *Saxo1*) were deleted in mice to determine their functional requirement in male fertility and potential utility as male contraceptive target. We chose to study these genes because all but *Saxo1* encode enzymes or enzyme-related protein products and are thus considered druggable in the classical sense. *Saxo1*, a cilia-related gene, was chosen because prior literature demonstrated expression in sperm [71]. Although not druggable in the classical sense, if targeted through non-canonical approaches, one could obtain a fast-acting drug with greater reversibility potential and a decreased likelihood of affecting testicular function and size. The epididymis-specific genes we chose to target for functional analysis, by the very nature of their tissue’s expression, also fit this potential drug profile of modulating only the latest stages of sperm development.

Analyses of testis and epididymis organ weights and histology, sperm parameters and morphology, and fertility revealed no significant differences in *Spint3*, *Pp2d1*, and *Saxo1* knockout mice in comparison to littermate controls demonstrating that, individually, *Spint3*, *Pp2d1*, and *Saxo1* are not required for male mouse fertility and are not suitable targets for the development of a male contraceptive. However, we found partial effects on male fertility in *Ces5a* knockout mice and profound effects on male fertility in *Spint4/5* double knockout mice.

CES5A is a member of a multigene family of mammalian carboxylesterases that can hydrolyze ester, thioester, amide, and carbamate linkages in a wide variety of endogenous and exogenous molecular substrates, including triglycerides, thus playing key roles in both metabolism and detoxification [72–75]. CES5A shares roughly similar percent homology (~ 40% homology) to all four of its related paralogs, CES1, CES2, CES3, and CES4A. Human carboxylesterase 1 (CES1) is predominantly expressed in the liver and has been shown to have triglyceride hydrolase activity as overexpression of human CES1 in cells leads to an increase in cholesteryl ester hydrolysis and free cholesterol efflux [76]. Further, mouse CES1G—a protein expressed by one of a cluster of eight syntenic genes (*Ces1a* through *Ces1h*) orthologous to the human *CES1* gene—has been shown to have triglyceride hydrolase activity as *Ces1g* null mice display hyperlipidemia and abnormal lipid homeostasis including increased liver and circulating cholesterol and triglycerides, and altered saturated and unsaturated fatty acid levels [77, 78]. Therefore, it is likely that CES5A exhibits similar carboxylesterase activity in the epididymis hydrolyzing cholesteryl ester and affecting free cholesterol efflux. Indeed, recombinant CES5A protein has been previously shown to have carboxylesterase activity hydrolyzing cholesterol ester and choline ester [79]. Since sperm cholesterol content is significantly decreased during epididymal maturation [80, 81] and a proper cholesterol/phospholipid (C/PL) ratio of the sperm plasma membrane is required for sperm capacitation [82, 83], CES5A may be pivotal in regulating sperm membrane cholesterol and lipid levels to ensure the normal function of male gametes in the last steps of the fertilization process.

The most closely related paralog to *SPINT4* is *EPPIN*, which, as reviewed in O’Rand et al., has at least three physiological functions [16]. EPPIN inhibits sperm motility when it binds the semen coagulation protein Semenogelin 1 (SEMG1) on the sperm surface [84]; it modulates the proteolytic activity of prostate-specific antigen (PSA), a serine protease, against its seminal plasma substrate, SEMG1 [85]; and it exhibits strong antibacterial activity [86]. These functions are postulated to prevent premature hyperactivation and capacitation of sperm in the female reproductive tract [16], and to protect spermatozoa from proteolytic and bacterial attack during transit in the female reproductive tract [11, 16]. Thus, it is possible that the physiological function of SPINT4 is similar. However, unlike SPINT1 and SPINT2, which have been shown to act as protease inhibitors against a wide variety of PRSS and TMPRSS proteases [87–90], when tested against a panel of eight proteases (including PSA, trypsin, chymotrypsin, plasmin, urokinase, thrombin, Factor Xa, and elastase), SPINT3 and SPINT4 were shown to lack protease inhibiting capability [91]. This indicates that either the protease inhibiting properties of SPINT3 and SPINT4 were lost in favor of yet unknown functions or their protease activity has a narrower spectrum of inhibition against unknown targets.

Since *Spint4/5* null male mice are severely subfertile, without an apparent difference in epididymis histology, sperm number, sperm morphology, and sperm motility parameters in comparison to the wild-type (WT) mice, this phenocopies the reproductive phenotype of several null mice of testis-, epididymis-, or prostate-specific genes (*Sof1*, *Tmem95*, and *Spaca6*; *Pate8*, and *Pate10*), which reveal a requirement in regulating sperm migration through the oviduct and sperm-oocyte fusion in mice [92, 93]. A severe fertility defect associated with normal sperm number, morphology, and motility is also shared among mice lacking the sperm membrane protein ADAM3, thought to be crucial in sperm-ZP binding and sperm migration through the uterotubular junction (UTJ) [94, 95]. More than 10 proteins including 2 proteases (ACE, ADAM1A, ADAM2, CALR3, CLGN, CMTM2A/B, PDILT, PMIS2, PRSS37, RNASE10, TEX101, and TPST2) have been described that affect the processing and/or localization of ADAM3 protein in spermatozoa [96, 97]. Further studies with *Spint4/5* null mice are required to determine whether sperm behavior in the female reproductive tract, specifically the sperm migration through the UTJ, is adversely affected. Since a large 16,797-bp genomic region—including the intergenic region between the *Spint4* and *Spint5* genes—was deleted (Fig. 8; Additional file 1: Table S12), based on the evidence presented in this manuscript, we cannot exclude the possibility that cis-acting elements and/or trans-acting factors affecting the expression of other genes may have contributed to the phenotype of these mice.

Lack of protein-coding ability of human *SPINT5P* does not necessarily indicate that this pseudogene is functionally obsolete. Pseudogenes have been shown to play roles in gene expression and gene regulation [98]. For example, pseudogene transcripts can act as competitive endogenous RNAs (ceRNA) through competitive binding of miRNA, which results in regulation of gene expression [99]. To this end, studying the functional requirement of *Spint5* in male mice is necessary to further our knowledge of evolutionarily conserved genes between species.

Since humans are genetically diverse, a limitation to phenotype characterization of genetically manipulated mice is the reliance of a single mouse background to the examination of complex genetic outcomes, such as fertility, that is under the control of many genes with different levels of contribution to the phenotype [100]. It is possible that a gene that causes complete infertility in an inbred mouse background may only cause partial infertility or subfertility in a different inbred line or more robust outbred background. Since the mice used in our study were a cross between C57BL/6 (B6) mice and DBA/2 (D2) mice, and thus, these B6D2F1 mice are heterozygous for B6 and D2 alleles at all loci in their genome, we can eliminate infertility susceptibility of either the B6 or the D2 background as the cause for fertility defects in *Ces5a* and *Spint4/5* mice. It does remain to be determined, however, whether the phenotype of the genes we knocked out would be more or less severe on a different mouse background, and if required for male fertility in humans, the level of contribution to male fertility of these genes across genetically diverse men.

A limitation to this study is the reliance on mRNA abundance positively correlating with protein abundance. Future studies are necessary to elucidate the relationship between mRNA and protein expression levels of the candidate genes identified in our study. Furthermore, despite batch corrections that were made, technical differences in sample preparation and integrity across the various published RNA-seq datasets can influence the results of our findings. One of the major advantages to our study design is the use of RNA-seq datasets from purified human and mouse germ cells and Sertoli cells to identify reproductive tract-specific targets since the use of whole testes for the identification of cell type-specific transcripts in past studies is subject to dilution effects. However, this advantage could also be considered a disadvantage since purified cells from non-reproductive tissues were not used for comparison but, if analyzed, purified cells from non-reproductive tissues may have revealed significant levels of expression in non-reproductive tissues. Ultimately, functional studies in animals and humans will help to confirm  whether genes identified in our study are essential for male fertility and not any other physiological process.

## Conclusions

Through the integration of hundreds of published and newly acquired human and mouse reproductive and non-reproductive tissue and cell RNA-seq datasets, we have generated a list of novel genes expressed predominantly or exclusively in the male reproductive tract that are worthy of consideration for functional validation in an animal model and potential targeting for a male contraceptive. Our results further validate a functional requirement for *Spint4/5* and *Ces5a* in male mouse fertility, while demonstrating that *Spint3*, *Pp2d1*, and *Saxo1* are each individually dispensable for male mouse fertility. Identifying novel reproductive tract-specific genes congruent across species adds insight into organismal biology and valuable information that can be used to identify potential male contraceptive drug target candidates. Furthermore, elucidating the individual functional requirement or lack thereof of these novel genes builds a better understanding of the factors underlying spermatogenesis and sperm maturation, which has implications in understanding the etiology of male infertility and further validation of the utility of a potential male contraceptive target.

## Materials and methods

### Human tissues and RNAs

The de novo isolated human testes and epididymides included in this study were obtained from three donors through a local organ transplant program in Quebec, Canada, called Transplant Quebec. All procedures were approved by the local ethics committee, and written consent was obtained from each respective donor’s family. The donors were of 40, 52, and 65 years of age with no preexisting medical condition that could affect reproductive function. Donor testes and epididymides were removed under artificial circulation to preserve other organs that were assigned for transplantation. Each testis and epididymis were dissected in the laboratory of Robert Sullivan at Université Laval. Each epididymis was dissected into three segments corresponding to the caput, corpus, and cauda regions and minced into small tissue pieces. Testes and epididymides tissue fragments were immediately snap frozen in liquid nitrogen, stored at − 80 °C, and shipped frozen to Baylor College of Medicine for further processing. Eight non-reproductive tissue types (kidney, liver, lung, skin, spleen, and stomach) and 2 female reproductive tissues (ovary and uterus) were obtained from the Baylor College of Medicine Tissue Acquisition and Pathology Core. Thirteen non-reproductive tissue types (adipose, adrenal gland, brain, colon, heart, leukocytes, pancreas, prostate, salivary gland, skeletal muscle, small intestine, smooth muscle, thyroid) were obtained as purified RNAs from Takara Bio (Kusatsu, Japan). Human testes and epididymis segments were used for de novo RNA-seq analysis; all human tissues and/or resulting RNAs were used for RT-PCR verification.

### Mouse tissues and RNAs

Mouse tissues [testis, caput, corpus, cauda, ovary, uterus, and 17 non-reproductive tissue types (adipose, bladder, brain, colon, eye, heart, kidney, liver, lung, prostate, skeletal muscle, skin, small intestine, spleen, stomach)] were obtained from dissection of B6/129 mice; the remaining 2 non-reproductive tissues (smooth muscle and thyroid) were obtained as purified RNAs from Takara Bio. Mouse epididymis segments were used for de novo RNA-seq analysis; all mouse tissues and/or resulting RNAs were used for RT-PCR verification.

### RNA isolation and reverse-transcription PCR

RNA for both RNA-seq and/or RT-PCR verification was isolated from human and mouse tissues using TRIzol/chloroform extraction method followed by RNeasy Mini kit from Qiagen with on-column DNase (Qiagen) treatment using the manufacturer’s protocol. RNAs used for RNA-seq were assessed by Bioanalyzer for RNA integrity. For RT-PCR, RNA was reverse-transcribed to cDNA using SuperScript III Reverse Transcriptase from Thermo Fisher according to the manufacturer’s protocol. cDNA was then PCR amplified using gene-specific primers designed using NCBI primer design tool*.* Primer sequences are listed in Additional file 1: Table S14.

### Library generation for RNA-seq

RNA-seq libraries were made using KAPA stranded mRNA-seq kit (KK8420). Briefly, Poly-A RNA was purified from total RNA using Oligo-dT beads; subsequently, it was fragmented to small size; and first strand cDNA was synthesized. Second strand cDNA was synthesized and marked with dUTP. Resultant cDNA was used for end repair, A-tailing, and adaptor ligation. Finally, the library was amplified for sequencing on an Illumina NovaSeq 6000 platform. The strand marked with dUTP was not amplified, allowing strand-specific sequencing.

### Sequence alignment, quantification, and differential gene expression

Human testes, human epididymis segments, and mouse epididymis segments were sequenced by the Department of Molecular and Human Genetics Functional Genomics Core at Baylor College of Medicine (Additional file 1: Table S1 and Additional file 1: Table S2). Previously published reproductive and non-reproductive tissue and cell sequences were downloaded from the Sequence Read Archive (SRA) [101] (Additional file 1: Table S1 and Additional file 1: Table S2). All sequences were trimmed using Trim Galore! and aligned against the human genome (GRCh38) or mouse genome (GRCm38) using HISAT2 [102, 103]. Gene expression in each tissue was quantified using featureCounts, filtered for only protein-coding genes, and batch corrected by removing unwanted variation using the RUVr method from RUVseq [104, 105]. Differential gene expression was determined for each reproductive tissue against each non-reproductive tissue using the R package EdgeR [106].

### Principal component analysis

Principal component analysis (PCA) was performed in the R statistical environment using the log2 counts per million (CPM) for each gene in the corresponding tissue after using the RUVr method to correct for batch variation as described above.

### Identification of male reproductive-specific gene drug candidates

Our procedure for identifying a reproductive-specific gene was repeated for each reproductive tissue or cell sample independently. The following selection criteria were applied to each reproductive tissue or cell sample. First, the non-reproductive tissue with the maximum expression, expressed as the log2 fold change between the non-reproductive tissue and the reproductive tissue or cell of interest, was identified for each gene using the results from the differential gene expression analysis. Second, we identified reproductive-specific gene drug candidates using three filters: a false discovery rate (FDR) filter, a maximum transcript per million (TPM) expression value filter on the non-reproductive sample with the maximum expression identified as described above, and a minimum TPM expression value filter on the reproductive tissue or cell sample of interest. A gene was kept if the FDR from the differential gene expression analysis was less than or equal to 0.05 for the comparison of the reproductive tissue or cell sample of interest to the non-reproductive tissue with the maximum expression. A gene was considered to be a male reproductive tissue-specific drug target if the average TPM expression value in the non-reproductive tissue with the maximum expression from the differential analysis was less than or equal to 1.0 for human (2.0 for mice), and if the average TPM expression value for that gene was greater than or equal to 10.0 for human (8.0 for mice) in the reproductive tissue or cell sample of interest. The average and standard deviation of the TPM expression value for each gene was calculated from the RUVr batch corrected counts per million expression value for each tissue or cell sample.

### Human to mouse gene symbol conversion

We consolidated data from Ensembl BioMart [29] and Mouse Genome Informatics (MGI) [28] to create a comprehensive database of mouse gene symbols orthologous to human genes and vice versa. Each respective species’ stable ensemble gene ID was used for each conversion, with gene symbol as the final output.

### Previously identified genes

As mentioned, several notable high-throughput gene expression studies using microarrays or RNA-seq, focused on identifying male reproductive tract-specific genes, have been previously published [2, 4–9]. Tables and Supplementary Tables from these studies were gathered to collect the lists of genes previously identified. For microarray-based studies, Affymetrix IDs were used to confirm the identity of a listed gene, based on current sequence mappings, or in many cases to identify de novo the identity of a gene only known at the time of the study by its Affymetrix ID and not gene symbol. For mouse and rat studies, gene symbols were converted to orthologous human symbols, to systematically catalog both the rodent and corresponding human symbols as previously identified. For example, 399 Affymetrix probe IDs were listed as reproductive tract-specific in Johnston et al. [8]. After re-identification of gene symbols based on current Ensembl sequence mappings, 42 identified gene symbols remained the same, 160 received an updated/replacement gene symbol identification, 103 genes that were previously unidentified received a new gene symbol identification, 26 Affymetrix IDs lost mapping to any gene symbol, and 67 remain unidentified. Out of the total of 305 Affymetrix IDs that mapped to 301 current rat gene symbols, 257 rat gene symbols converted to at least one human ortholog gene symbol that was either the same symbol or different. Both rat and human symbols, based on new mappings, were considered previously identified. For the complete list of previously identified genes, see Additional file 18: Table S10.

### Drug target type classification

Genes were classified as encoding either enzymes, epigenetic-related proteins, G protein-coupled receptors (GPCRs), ion channels, kinases, nuclear receptors, orphan GPCRs (oGPCRs), transcription factors, transporters, or unknown proteins based on data obtained from Illuminating the Druggable Genome [27] (Additional file 11: Table S8).

### Availability of a mouse model

We used data obtained from Ensembl BioMart [29], MGI [28], the International Mouse Phenotyping Consortium (IMPC) [31], and PubMed searches to generate a comprehensive database identifying the existence of a mouse model for all mouse genes (Additional file 12: Table S9). We then queried our identified candidate human and mouse genes against this list. For a given human gene, we queried the equivalent mouse ortholog gene symbol(s).

### Experimental animals

*Spint3*, *Spint4/5*, *Ces5a*, *Pp2d1*, and *Saxo1* knockout mice were produced at Baylor College of Medicine. B6D2F1 (C57BL/6 × DBA2) mice were used as embryo donors, and CD1 mice were used as foster mothers. Mice were purchased from Charles River (Wilmington, MA). All mice were housed in a temperature-controlled environment with 12-h light cycles and free access to food and water. Mice were housed in accordance with NIH guidelines, and all animal experiments were approved by the Institutional Animal Care and Use Committee (IACUC) at Baylor College of Medicine.

### Generation of *Spint3*, *Spint4/5*, *Ces5a*, *Pp2d1*, and *Saxo1* knockout mice

To generate *Spint3*, *Spint4/5*, *Ces5a*, *Pp2d1*, and *Saxo1* knockout mice, gRNA/Cas9 ribonucleoprotein complex was electroporated into fertilized eggs and transplanted into surrogate mothers as previously described [107]. Briefly, to harvest fertilized eggs, CARD HyperOva (0.1 mL, Cosmo Bio) was injected into the abdominal cavity of B6D2F1 females (Charles River), followed by human chorionic gonadotropin (hCG) (5 units, EMD Chemicals). Forty-eight hours after CARD HyperOva, B6D2F1 males were allowed to mate naturally. Twenty hours after mating, fertilized eggs with 2 pronuclei were collected for electroporation. Custom crRNAs targeting each gene were purchased from Millipore-Sigma. The sequences for all guide RNAs used for CRISPR/Cas9-mediated gene editing are listed in Additional file 1: Table S11. crRNA and tracrRNA (Millipore-Sigma) were diluted with nuclease-free water. The mixture was denatured at 95 °C for 5 min and allowed to anneal by cooling gradually to room temperature (1 h). Each gRNA was mixed with Cas9 protein solution (Thermo Fisher Scientific) and opti-MEM media (Thermo Fisher Scientific), and then incubated at 37 °C for 5 min to prepare the gRNA/Cas9 RNPs [final concentration, 300 ng/μL Cas9 for 250 ng/μL of each gRNA]. The gRNA/Cas9 RNP solution was placed between electrodes with a 1-mm gap in the ECM 830 Electroporation System (BTX). Fertilized eggs were arranged between the electrodes, and then, the electroporation was performed with the following conditions: 30 V, 1-ms pulse duration, and 2 pulses separated by 100-ms pulse interval. For egg transfer, electroporated embryos were transplanted into the oviduct of pseudo-pregnant ICR recipients. After 19 days, offspring were obtained by natural birth or Cesarean section. The F0 mice with sequence-predicted heterozygous mutations were used for the mating with additional B6D2F1 mice to generate homozygous mutants. The F2 or later generations were used for the phenotypic analyses.

### Genotype analysis of knockout mice

For Sanger sequence analysis of mutant mice, genomic DNA was isolated by incubating tail tips in lysis buffer [20 mM Tris-HCl (pH 8.0), 5 mM EDTA, 400 mM NaCl, 0.3% SDS, and 200 μg/mL Actinase E solution] at 60 °C overnight. Polymerase chain reactions (PCRs) amplifying the genomic region containing the insertion/deletion events were performed using KOD Xtreme enzyme (TOYOBO, Osaka, Japan); PCR products were purified using the QIAquick PCR Purification Kit (Qiagen, Carlsbad, CA, USA) and sent for Sanger sequencing on an ABI 3130XL Genetic Analyzer (Thermo Fisher Scientific, Waltham, MA, USA) using the forward primer. For routine genotyping of mutant mice, genomic DNA was isolated by separately incubating ear snips and tail tips in 50 mM NaOH solution at 95 °C overnight and inactivating with 1 M Tris pH = 8.0. PCRs amplifying wild-type and mutant-specific amplicons were performed using 2X amfiSure PCR Master Mix (GenDEPOT, Barker, TX). Primer sequences are listed in Additional file 1: Table S13.

### Male fertility analysis

Upon sexual maturation (6–7 weeks of age), knockout and littermate control male mice (*n* = 6–9 mice per genotype) were continuously housed with two 7–8-week-old wild-type B6D2F1/J female mice per male for 12 weeks. During the fertility test, the number of pups was counted shortly after birth. The total number of litters and pups per male over the mating trial was calculated and divided by the number of months to generate averages and statistics per genotype. The average number of pups per litter is based on the average litter size per male where a litter is considered one or more pups.

### Testis weights and testis and epididymis histology of male mice

Knockout and littermate control male mice (*n* = 5–13 mice per genotype) that were 12 weeks of age were used to examine body and reproductive organ weights, and testicular and epididymal histology. Testes and epididymides were fixed in Bouin’s fixative, embedded in paraffin, sectioned at 5 μm thickness, and stained with 1% periodic acid-Schiff (PAS) stain followed by counterstaining with hematoxylin 2 solution. Histological images were acquired with an Aperio AT2 slide scanner (Leica Microsystems).

### Sperm analysis

Knockout and littermate control male mice (*n* = 5–13 mice per genotype) that were 12 weeks of age were used to examine sperm numbers and motility parameters using computer-assisted sperm analysis (CASA). Cauda of both epididymides was isolated, transferred into Human Tubule Fluid (HTF) (Irvine Scientific, Santa Ana, CA) containing 5 mg/mL of BSA, minced, and placed in a humidified incubator for 15 min at 37 °C with 5% CO_2_. Following incubation, the sperm were diluted 1:50 in HTF, added to a pre-warmed slide, and analyzed using a Hamilton-Thorne Bioscience’s Ceros II instrument. Several fields of view were illuminated and captured until at least 200 cells were counted.

### RNA in situ hybridization

RNAscope 2.5 HD Reagent Kit (RED) (cat. 322350, Advanced Cell Diagnostics, Newark, CA, USA) was used to detect *Spint3*, *Spint4*, and *Spint5* mRNA transcripts on PFA-fixed, paraffin-embedded sections from 3-month-old wild-type epididymis. The probes against Mm-*Spint3*, Mm-*Spint4*, and Mm-*Spint5* were custom-made, and the standard positive control (Mm-*Ppib*, cat. 313911) and negative control (DapB, cat. 310043) probes were used. The assay was performed according to the manufacturer’s instructions. Slides were counterstained using DAPI and mounted using ProLong Glass Antifade Mountant (Thermo Fisher Scientific Inc.). Multi-channel fluorescent images were acquired with an Aperio VERSA (Leica Microsystems).

### Statistical analysis

All measurements are expressed as mean ± standard error of the mean. Statistical differences were determined using Student’s *t* test. Differences were considered statistically significant if the *P* value was less than 0.05.

## Supplementary information

**Additional file 1: Tables S1, S2, S11, S12, S13, and S14. Table S1.** Summary of Human RNA-seq datasets. This table contains the SRA value for each previously published human RNA-seq dataset that was reanalyzed as part of this study. The GEO accession number for each new human RNA-seq dataset generated and subsequently analyzed in this study is also included. **Table S2.** Summary of Mouse RNA-seq datasets. This table contains the SRA value for each previously published mouse RNA-seq dataset that was reanalyzed as part of this study. The GEO accession number for each new mouse sample generated and subsequently analyzed in this study is also included. **Table S11.** Single-guide RNAs targeting the genes’ upstream (U) and downstream (D) regions used for generating knockout mice. Efficiency of embryo transplantation was presented using the number of total pups delivered by pseudopregnant mice divided by the number of total embryos used for oviduct transplantation (Total pups/embryos transplanted). Efficiency of genome editing was determined by the number of pups carrying enzymatic mutations divided by the number of pups subjected to genotyping (GM pups/pups genotyped). **Table S12.** Sanger sequencing of detailed genotype of mutant DNA sequences in all the five mouse lines. **Table S13.** Primers and PCR conditions used for genotyping the mutant alleles of the knockout mouse lines. **Table S14.** Human and mouse RT-PCR primer sequences used for verification of reproductive tract-specificity.

**Additional file 2: Fig. S1.** Genes that passed the TPM and FDR filters in at least one of the measured reproductive tissues or cells were visualized using a heatmap of the RUVr batch corrected log2 CPM gene expression values for the human **(A)** and mouse **(B)** samples.

**Additional file 3: Table S3.** Human Expression Summary. Contains differential fold change, identity of the non-reproductive tissue with maximal gene expression based on the differential gene analysis, false detection rate (FDR) value, average and standard deviation TPM expression values, and log2 CPM gene expression value for the human samples. All protein-coding genes (18,305 genes) that had expression in at least one reproductive tissue or cell is listed.

**Additional file 4: Table S4.** Mouse Expression Summary. Contains differential fold change, identity of the non-reproductive tissue with maximal gene expression based on the differential gene analysis, false detection rate (FDR) value, average and standard deviation TPM expression values, and log2 CPM gene expression value for the mouse samples. All protein-coding genes (16,891 genes) that had expression in at least one reproductive tissue or cell is listed.

**Additional file 5: Table S5.** All human male reproductive tract-specific genes that met the criteria of identification as reproductive tract-specific in at least one male reproductive tissue or purified cell type, with the level of fold change listed under the tissue or cell if all criteria were met. The criteria of selection are as follows: FDR < 0.05; TPM_repro_ > 10; TPM_non-repro_, max < 1. A fold change value of 0 indicates the criteria were not met for that that tissue or cell. Additional columns indicating 1.) the equivalent mouse ortholog gene symbols (single or multiple symbols) that exist, and 2.) if our studies identified any of these mouse orthologs as reproductive tract-specific in mouse, are included.

**Additional file 6: Table S6.** All mouse male reproductive tract-specific genes that met the criteria of identification as reproductive tract-specific in at least one male reproductive tissue or purified cell type, with the level of fold change listed under the tissue or cell if all criteria were met. The criteria of selection are as follows: FDR < 0.05; TPM_repro_ > 8; TPM_non-repro_, max < 2. A fold change value of 0 indicates the criteria were not met for that that tissue or cell. Additional columns indicating 1.) the equivalent human ortholog gene symbols (single or multiple symbols) that exist, and 2.) if our studies identified any of these human orthologs as reproductive tract-specific in human, are included.

**Additional file 7: Fig. S2.** Summary of number of statistically significant up and down-regulated genes, and quantification of candidate genes with respect to the individual reproductive tissue or cell of interest. The plots in panels **(A)** and **(B)** summarizes the number of statistically significant human or mouse genes respectively, that are up-regulated or down-regulated in each reproductive tissue or cell of interest compared to the non-reproductive tissue with maximal gene expression. Red columns depict the number genes that are up-regulated and blue columns depict the number genes that are down-regulated. Changes in gene expression were considered statistically significant for an FDR of less than or equal to 0.05. The total number of candidate genes are designated by the black columns. Candidate genes are genes that passed the FDR and TPM expression value filters.

**Additional file 8: Fig. S3.** Venn diagrams comparing the overlap between the candidate male reproductive genes identified by the indicated reproductive tissues. The human testis combined gene list is the list of genes from both new samples we isolated and from previously published testis samples. The human epididymis combined gene list is the list of genes identified in either previously published samples or the newly generated samples across all sections of the epididymis. Lastly, the mouse epididymis combined gene list is the list combined list of genes identified across all three sections of the mouse epididymis.

**Additional file 9: Table S7**. Complete cross-sample comparison identifying human and mouse reproductive tract specific genes common to two or more samples and unique to each as identified through our studies.

**Additional file 10: Fig. S4.** Classification of genes into different protein families and identification of the existence of an experimental mouse model. Each candidate human gene was classified as an enzyme (enzyme), chromosome and histone modifiers (epigenetic), G-protein-coupled receptor (GPCR), orphan G-protein-couple receptor (oGPCR), kinase (kinase), transcription factor (TF), nuclear receptor (NR), ion channel (IC), chromosome and histone modifying transcript factor (TF; epigenetic), transporter (transporter) and unknown (A). The total number of candidate genes identified in our search for mouse models were plotted. Orange columns designate the number of candidate genes where a model was identified while yellow designates candidate genes where a model was not identified (B).

**Additional file 11: Table S8.** Drug Target Type Classification for Human Genes. Genes are listed according to the tissue and/or cell that they were identified as reproductive tract-specific in.

**Additional file 12: Table S9.** Availability of a Mouse Model for Human Genes with a Mouse Ortholog. Genes are listed according to the tissue and/or cell that they were identified as reproductive tract-specific in.

**Additional file 13: Fig. S5.** One-hundred and forty-two previously identified human male reproductive tract-specific genes that remain without a reported mouse model. The listed genes were identified in one or more datasets as indicated in the Venn diagram. Underlined genes were also identified in our studies as reproductive tract-specific in mouse. Genes written in blue encode either enzymes, kinases, GPCRs, oGPCRs, transporters, transcription factors, or proteins involved in epigenetic regulation. Genes written in dark red were identified in both testis (testis and/or testis cell) and in epididymis.

**Additional file 14: Fig. S6.** Seventy-three human male reproductive tract-specific genes that each have a reported mouse model with male infertility phenotype. The listed genes were identified in one or more datasets as indicated in the Venn diagram. Underlined genes were also identified in our studies as reproductive tract-specific in mouse. Genes written in blue encode either enzymes, kinases, GPCRs, oGPCRs, transporters, transcription factors, or proteins involved in epigenetic regulation. Genes written in dark red were identified in both testis (testis and/or testis cell) and in epididymis.

**Additional file 15: Fig. S7.** RT-PCR confirmation of reproductive tract-specificity in both humans (A) and mice (B). The genes listed in this figure were identified through our studies and previous studies, but currently remain without a reported mouse model. *GAPDH* and *Hprt* are included as housekeeping genes.

**Additional file 16: Fig. S8.** Eighty-nine novel human genes without a mouse ortholog. The listed genes were identified in one or more datasets as indicated in the Venn diagram. Genes written in blue encode either enzymes, kinases, GPCRs, oGPCRs, transporters, transcription factors, or proteins involved in epigenetic regulation. Genes written in dark red were identified in both testis (testis and/or testis cell) and in epididymis.

**Additional file 17: Fig. S9.** Novel reproductive tract-specific human genes that do not have any equivalent mouse orthologs. These genes may serve as potential contraceptive targets, however functional validation would need to be carried out in another model organism than mouse, such as rat or marmoset, which do have orthologs to these genes. The digital PCR (heatmap) depicts the average transcripts per million (TPM) value per tissue per gene from the indicated human RNA-seq datasets as processed in parallel through our bioinformatics pipeline. White = 0 TPM, Black ≥30 TPM. The expression profile of the human housekeeping gene, *GAPDH*, is included as reference. For data obtained from published datasets, superscript values reference the dataset publication as previously mentioned.

**Additional file 18: Table S10.** 1064 Previously Identified Genes. Genes previously identified as male reproductive tract-specific through high throughput gene expression studies using either microarrays or RNA-seq [2–8]. The human ortholog to genes identified in mouse and rat studies is included.

**Additional file 19: Fig. S10.** Developmental expression pattern of *Spint3*, *Spint4*, *Spint5*, *Pp2d1*, and *Saxo1* in epididymis and testis of postnatal and adult mice. Whole epididymides were used at postnatal days 3, 6, 10, and 14 and epididymis segments (caput, corpus, and cauda) were used at postnatal days 21, 28, 35, and 60. Whole testes were used at all time points. The housekeeping gene, *Hprt*, was used as reference.

**Additional file 20: Fig. S11.** Multi-channel fluorescence images of bilateral epididymis serial sections stained with custom RNAscope probes targeting either *Spint3*, *Spint4*, or *Spint5* mRNA (red) and DAPI (blue). The position of Caput (Cap), Corpus (Cor), and Cauda (Cau) is labeled in the overview image (left column). The position of the magnification over the epididymis is the same for all three sections (right column).

**Additional file 21: Fig. S12.** Representative periodic acid-Schiff staining of *Spint3* and *Spint4/5* knockout and littermate control (wild-type) testes and epididymis segments (caput, corpus, and cauda) at 3 months of age.

**Additional file 22: Fig. S13.** Representative periodic acid-Schiff staining of *Ces5a* knockout and littermate control (wild-type) testes and epididymis segments (caput, corpus, and cauda) at 3 months of age.

**Additional file 23: Fig. S14.** Representative scanning electron microscopy images of *Spint4/5* and *Ces5a* KO and littermate control mouse sperm.

**Additional file 24: Fig. S15.** Representative periodic acid-Schiff staining of *Pp2d1* and *Saxo1* knockout and littermate control (wild-type) testes and epididymis segments (caput, corpus, and cauda) at 3 months of age.

## Data Availability

All data generated or analyzed during this study are included in this published article, its supplementary information files, and publicly available repositories. The SRA values for each of the 162 previously published reproductive and non-reproductive human RNA-seq datasets [9, 21, 23, 24, 26, 30] and 81 previously published reproductive and non-reproductive mouse RNA-seq datasets [19, 22, 25] are listed in Additional file 1: Table S1 and Additional file 1: Table S2. All raw and processed data for the 12 new human and 9 new mouse samples generated in this study is deposited in NCBI GEO (Accession GSE150854). All mice generated in this study, and any additional information about this study, are available from the corresponding authors upon request.
