# Supplementary material for: Large-scale discovery of male reproductive tract-specific genes through analysis of RNA-seq datasets
Source: BMC Biol. 2020 Aug 19;18:103. doi: 10.1186/s12915-020-00826-z (PMC7436996; doi:10.1186/s12915-020-00826-z)
Supplement: Supplementary file 1 — Additional file 1: Tables S1, S2, S11, S12, S13, and S14. Table S1. Summary of Human RNA-seq datasets. This table contains the SRA value for each previously published human RNA-seq dataset that was reanalyzed as part of this study. The GEO accession number for each new human RNA-seq dataset generated and subsequently analyzed in this study is also included. Table S2. Summary of Mouse RNA-seq datasets. This table contains the SRA value for each previously published mouse RNA-seq dataset that was reanalyzed as part of this study. The GEO accession number for each new mouse sample generated and subsequently analyzed in this study is also included. Table S11. Single-guide RNAs targeting the genes’ upstream (U) and downstream (D) regions used for generating knockout mice. Efficiency of embryo transplantation was presented using the number of total pups delivered by pseudopregnant mice divided by the number of total embryos used for oviduct transplantation (Total pups/embryos transplanted). Efficiency of genome editing was determined by the number of pups carrying enzymatic mutations divided by the number of pups subjected to genotyping (GM pups/pups genotyped). Table S12. Sanger sequencing of detailed genotype of mutant DNA sequences in all the five mouse lines. Table S13. Primers and PCR conditions used for genotyping the mutant alleles of the knockout mouse lines. Table S14. Human and mouse RT-PCR primer sequences used for verification of reproductive tract-specificity. [file 12915_2020_826_MOESM1_ESM.docx]

*Robertson et al.* **Tables S1, S2, S11, S12, S13, and S14**

**Table S1.** Summary of Human RNA-seq datasets. This table contains the SRA value for each previously published human RNA-seq dataset that was reanalyzed as part of this study. The GEO accession number for each new human RNA-seq dataset generated and subsequently analyzed in this study is also included.

| **Tissue** | **Samples (N)** | **SRA Run #** |
| --- | --- | --- |
| adipose tissue | 5 | ERR315332, ERR315343, ERR315342, ERR315378, ERR315431 |
| adrenal gland | 5 | ERR315335, ERR315392, ERR315417, ERR315385, ERR315452 |
| appendix | 5 | ERR315345, ERR315366, ERR315437, ERR315465, ERR315481 |
| bladder | 5 | ERR315334, ERR315370, ERR315355, ERR315447, ERR315421 |
| bone marrow | 5 | ERR315333, ERR315395, ERR315425, ERR315396, ERR315406 |
| colon | 5 | ERR579129, ERR315348, ERR315357, ERR315403, ERR315400 |
| cerebral cortex | 3 | ERR315432, ERR315455, ERR315477 |
| duodenum | 4 | ERR315442, ERR315445, ERR315457, ERR315461 |
| heart | 5 | ERR315328, ERR315331, ERR315384, ERR315367, ERR315356 |
| gallbladder | 5 | ERR315349, ERR315347, ERR315360, ERR315427, ERR315474 |
| esophagus | 5 | ERR315362, ERR315434, ERR315398, ERR315411, ERR315472 |
| kidney | 4 | ERR315383, ERR315443, ERR315468, ERR315494 |
| liver | 5 | ERR315327, ERR315414, ERR315394, ERR315463, ERR315451 |
| lung | 5 | ERR315326, ERR315341, ERR315346, ERR315353, ERR315439 |
| lymph node | 5 | ERR315329, ERR315373, ERR315371, ERR315426, ERR315393 |
| pancreas | 4 | ERR315429, ERR315436, ERR315466, ERR315479 |
| prostate | 5 | ERR315330, ERR315340, ERR315359, ERR315365, ERR315354 |
| rectum | 4 | ERR579140, ERR579151, ERR579147, ERR579127 |
| salivary gland | 5 | ERR315325, ERR315382, ERR315420, ERR315418, ERR315459 |
| skeletal muscle | 3 | ERR579142, ERR579143, ERR579149 |
| skin | 5 | ERR315339, ERR315372, ERR315376, ERR315401, ERR315460 |
| small intestine | 5 | ERR315344, ERR315381, ERR315364, ERR315409, ERR315419 |
| spleen | 5 | ERR315338, ERR315416, ERR315448, ERR315405, ERR315473 |
| stomach | 4 | ERR315369, ERR315379, ERR315485, ERR315467 |
| thyroid gland | 5 | ERR315337, ERR315358, ERR315363, ERR315428, ERR315397 |
| tonsil | 2 | ERR579133, ERR579139 |
| testis | 5 | ERR315351, ERR315352, ERR315350, ERR315391, ERR315446 |
| cauda | 2 | SRR2392510, SRR2392509 |
| corpus | 2 | SRR2392508, SRR2392507 |
| caput | 2 | SRR2392506, SRR2392505 |
| testis | 3 | New samples; GEO: GSM4559718, GSM4559721, GSM4559726 |
| caput | 3 | New samples; GEO: GSM4559719, GSM4559720, GSM4559728 |
| corpus | 3 | New samples; GEO: GSM4559724, GSM4559725, GSM4559729 |
| cauda | 3 | New samples; GEO: GSM4559722, GSM4559723, GSM4559727 |
| spermatogonial stem cells (SSEA4-/KIT+) | 6 | SRR5099528, SRR5099527, SRR5099518, SRR5099517, SRR5099516, SRR5099515 |
| spermatogonial stem cells (SSEA4+/KIT-) | 4 | SRR5099526, SRR5099525, SRR5099520, SRR5099519 |
| spermatogonial stem cells (SSEA4+) | 6 | SRR5099524, SRR5099523, SRR5099514, SRR5099513, SRR5099512, SRR5099511 |
| spermatogonial stem cells (KIT+) | 2 | SRR5099522, SRR5099521 |
| sertoli cells | 3 | SRR3538834, SRR3538932, SRR3538933 |
| primary spermatocytes | 3 | SRR3543511, SRR3543512, SRR3543513 |
| spermatids | 3 | SRR3543514, SRR3543515, SRR3543516 |
| undifferentiated spermatogonia | 3 | SRR3543517, SRR3543518, SRR3543519 |
| sertoli cells (mock zika) | 3 | SRR5251504, SRR5251503, SRR5251502 |

**Table S2.** Summary of Mouse RNA-seq datasets. This table contains the SRA value for each previously published mouse RNA-seq dataset that was reanalyzed as part of this study. The GEO accession number for each new mouse sample generated and subsequently analyzed in this study is also included.

| **Tissue** | **Samples (N)** | **SRA Run #** |
| --- | --- | --- |
| adipose tissue | 4 | SRR5048011, SRR5048013, SRR5048012, SRR5048014 |
| adrenal gland | 5 | SRR5047957, SRR5047958, SRR5047959, SRR5047960, SRR5047961 |
| bone marrow | 2 | SRR5171014, SRR5171015 |
| colon | 5 | SRR5047913, SRR5047914, SRR5047915, SRR5047916, SRR5047917 |
| cerebral cortex | 4 | SRR3191882, SRR3191883, SRR3191884, SRR3191885 |
| duodenum | 5 | SRR5047963, SRR5047964, SRR5047965, SRR5047966, SRR5047967 |
| heart | 5 | SRR5047921, SRR5047922, SRR5047923, SRR5047924, SRR5171028 |
| kidney | 5 | SRR5047925, SRR5047926, SRR5047927, SRR5047928, SRR5047929 |
| liver | 5 | SRR5047931, SRR5047932, SRR5047933, SRR5047934, SRR5047935 |
| lung | 5 | SRR5047937, SRR5047938, SRR5047939, SRR5047940, SRR5171030 |
| pancrease | 2 | SRR5171086, SRR5171087 |
| skeletal muscle | 5 | SRR3191889, SRR3191890, SRR3191891, SRR3191892, SRR3191893 |
| small intestine | 5 | SRR5048010, SRR5171046, SRR5171047, SRR5171080, SRR5171081 |
| stomach | 5 | SRR5047995, SRR5047996, SRR5047997, SRR5047998, SRR5047999 |
| testis | 6 | SRR5047953, SRR5047954, SRR5047955, SRR5047956, SRR5171084, SRR5171085 |
| caput | 3 | New samples; GEO: GSM4559730, GSM4559731, GSM4559732 |
| corpus | 3 | New samples; GEO: GSM4559736, GSM4559737, GSM4559738 |
| cauda | 3 | New samples; GEO: GSM4559733, GSM4559734, GSM4559735 |
| high-ID4_spermatogonia* | 3 | SRR5189921, SRR5189922, SRR5189923, SRR5189927, SRR5189928, SRR5189929, SRR5189933, SRR5189934, SRR5189935 |
| low-ID4_spermatogonia* | 3 | SRR5189924, SRR5189925, SRR5189926, SRR5189936, SRR5189937, SRR5189938, SRR5189930, SRR5189931, SRR5189932 |
| 35d-Sox9_sertoli | 3 | SRR1523341, SRR1523340, SRR1523342 |
| spermatogonia‡ | 1 | SRR3437699 |
| leptotene spermatocytes‡ | 1 | SRR3437700 |
| pachytene spermatocytes‡ | 1 | SRR3437701 |
| round spermatids‡ | 1 | SRR3437702 |

* Technical replicates were merged to form three biological replicates.

‡ results of reanalysis included in dPCR, but not in fold-change and FDR calculations

**Table S11.** Single-guide RNAs targeting the genes’ upstream (U) and downstream (D) regions used for generating knockout mice. Efficiency of embryo transplantation was presented using the number of total pups delivered by pseudopregnant mice divided by the number of total embryos used for oviduct transplantation (Total pups/embryos transplanted). Efficiency of genome editing was determined by the number of pups carrying enzymatic mutations divided by the number of pups subjected to genotyping (GM pups/pups genotyped).

| **Gene** | **KO strategy** | **Total pups/embryos transplanted** | **GM pups/ pups genotyped** | **sgRNA sequences** | | **PAM** |
| --- | --- | --- | --- | --- | --- | --- |
| *Spint3* | Zygote electroporation | 25/78 (32%) | 13/25 (52%) | U | GGAACAAGAAGAAGTAGGCC | TGG |
|  |  |  |  | D | TTGGTTCTGCGCAAAGCTCT | TGG |
| *Spint4/5* | Zygote electroporation | 19 | 19/19 (100%)* | U | AGGAGAAATCCCAGCTTAGT | GGG |
|  |  |  |  | D | CACAGGGCAAGGCGAATCCC | TGG |
| *Ces5a* | Zygote electroporation | 19/54 (35%) | 12/19 (63%) | U | ATCCCAAGAAACACGTTCAC | GGG |
|  |  |  |  | D | GCCACAGCCTCCGATCCCCT | GGG |
| *Pp2d1* | Zygote electroporation | 19/77 (25%) | 13/19 (68%) | U | TTCACGGTCGTGAACGATTT | TGG |
|  |  |  |  | D | ATTCCTTTGACACGGTGTTC | AGG |
| *Saxo1* | Zygote electroporation | 7/40 (18%) | 5/7 (71%) | U | TAAATCTTCGTAGGGAGATG | TGG |
|  |  |  |  | D | CTGATAGATCGGATAATTCT | CGG |

**Table S12.** Sanger sequencing of detailed genotype of mutant DNA sequences in all the five mouse lines.

| **Gene** | **Upstream sequence (25 bp)** | **Mutation** | **Downstream sequence (25 bp)** |
| --- | --- | --- | --- |
| *Spint3* | TGGCAGACACCATGCAGCTCCAGGC | -41 | TTTGCGCAGAACCAAATAATGGTAG |
| *Spint4/5* | TGCTTGCTCCACCATGAAGCCCACT | -16,797 | ATTCGCCTTGCCCTGTGGCTTCTAA |
| *Ces5a* | TGCGGTTTTCCAAGCCACAGCCTCC | -5 | CCTGGGATAATTTGCGAGAAGCCAC |
| *Pp2d1* | CGGAGCCGAACTGTAAATTCACGGT | -16 | GATAAGGCCAATGTGTGCTTTTTCG |
| *Saxo1* | ATCTTTTGTAGGCGGCATCGCTGTC | -77 | TATCAGTCTTACCTGCCCAGAAATT |

**Table S13.** Primers and PCR conditions used for genotyping the mutant alleles of the knockout mouse lines.

| **Gene** | **Allele** | **Forward primer** | **Reverse primer** | **Size (bp)** | **Annealing (°C)** | **Elongation  (sec)** |
| --- | --- | --- | --- | --- | --- | --- |
| *Spint3* | WT | TCCTCATCTTCTGCCAAGAGC | ACCCATTTTCCCACGAGGTC | 325 | 60 | 30 |
|  | KO | GGCAAACTGGGGCTGAAAAG | TTTGGTTCTGCGCAAAGCCTG | 457 | 60 | 30 |
| *Spint4/5* | WT | GCTTTTCAGAACACGGGAAAGAAGCG | CTCTCTCTGGAAAGGTCTTACTTGTGCC | 630 | 60 | 30 |
|  | KO | CTCCAATGGTCCAACCCTCC | GTTGGTCGTCAGCCATACCA | 483 | 60 | 30 |
| *Ces5a* | WT | ATCAGGGGCCTGTCTAGGTT | CGCAAATTATCCCAGGGGATC | 599 | 60 | 30 |
|  | KO | ACCCCGTAATCCATTTGGCA | GCAAATTATCCCAGGGGAGG | 459 | 60 | 30 |
| *Pp2d1* | WT | ACGGTCGTGAACGATTTTGGT | TACAAGTGAGTGCCGAGCAG | 388 | 60 | 30 |
|  | KO | ACCAAAGCCCAAACTGGTCA | ACACATTGGCCTTATCACCGT | 289 | 60 | 30 |
| *Saxo1* | WT | CGTCTTCTCTCCGAGTACACC | ACTGCACGCTGTTACCTTGA | 209 | 60 | 30 |
|  | KO | TCGCAGAAGAGTTGGACACC | GCAGGTAAGACTGATAGACAGCG | 348 | 60 | 30 |

**Table S14.** Human and mouse RT-PCR primer sequences used for verification of reproductive tract-specificity.

| **Gene** | **Species** | **Forward Primer** | **Reverse Primer** | **Size (bp)** | **Cycles** |
| --- | --- | --- | --- | --- | --- |
| *AC022167.5* | Human | AATCATCCCCGGCTGTATGC | ACGAGTGCTTCACGTCCATT | 220 | 30 |
| *AC187653.1* | Human | AGGTCCCTGAGTGCAAACTG | TCCGCTATACCCAGCAAGTC | 70 | 30 |
| *ADAM20* | Human | AGGGTCATGCCCACATCAAT | TGCAGTGCTGAAAATAAAAACTCTG | 213 | 30 |
| *AL672043.1* | Human | CCCACCTCAAGTTCTCAGACATC | TTGTTGTTCACCACCCACAC | 135 | 30 |
| *BHMG1* | Human | CCGGTTGGGAACGTGGTGT | GCTGGAACCAAACATCTCAGGG | 174 | 30 |
| *BSPH1* | Human | CTGTGAATGATGGTTTGCTTGC | AGACCATTCTGACCTGCTGAA | 123 | 30 |
| *BX276092.9* | Human | CACCTGATCCGGCTAGCTTC | CTGGCTCCAACTTCACAGGTC | 515 | 30 |
| *C16orf95* | Human | AGAAGCCATATGCGTGCGAG | TTGAACCGTCTTTTGACTCTGCT | 357 | 30 |
| *C1orf105* | Human | AAAAACACTTTGGATTCAGGTTCTC | TCAAATTTTGGAACAGAAGCCTTTA | 155 | 30 |
| *C2orf92* | Human | GAGCCATGGCCCTCTTCTTT | GCCAGAGGAGATGATGGCTG | 670 | 30 |
| *C4orf51* | Human | GCATGGAGTGGCACATCAAAT | GCTTTCAGAATCGCACCGTC | 172 | 30 |
| *CCDC196* | Human | AGGATCTTACCTGCCCTCAGA | GCCCATAGTACCTTGGGACC | 703 | 30 |
| *CES5A* | Human | CTGCCTCTGCCTGCCTATTC | CAGAAGGCCCTTTGGTGGG | 203 | 30 |
| *CPA5* | Human | CCTTCAAACCCTGTGAGGCT | GCCATAAGCGGTTCATGCTG | 870 | 30 |
| *DCAF8L1* | Human | CAGTCCAGCTAGATGCCACC | TGTGTATGCACTCATATAAGGGGT | 131 | 30 |
| *EFCAB5* | Human | GCAGGAGTGAGGGAGCTTTT | GGCACACTCTGTAAGGTCTCA | 253 | 30 |
| *ERICH6* | Human | GACTACGACGACGACTTCCC | GCTCCTGTTTCCTTTGCAGG | 796 | 30 |
| *ETDB* | Human | GGAAAGAGAGGGAGTGCCAAG | AGGAAGTCTGGGATATGGGC | 327 | 30 |
| *FER1L5* | Human | GCCTAAACGGTATGAGCTGC | GCTCATGGCCAAATAGTGCG | 817 | 30 |
| *GLT6D1* | Human | GCCCTGCAGTGGAGAGATTT | CCTCTGCAAACCTGCCAGTA | 470 | 30 |
| *IQCA1L* | Human | TCGAGTGCCATGACCTTACTG | AAGGACCGCATACAAAGCCA | 725 | 30 |
| *LCN6* | Human | CCAGAAAACAACCTGCGGAC | GCACGCCTATTGAGGGATTCT | 121 | 30 |
| *MAGEA11* | Human | AGCGAAAGCGTCTTTCTGAGG | AGAACATAGTGCTCACCTGGAGTC | 203 | 30 |
| *MAGEB6* | Human | TTCTCAGCTGTCTTGGTGCCAG | GGCTTGTACTCTCTGCGGAC | 768 | 30 |
| *MROH2B* | Human | ATCTACCAAGGGCCAGGGAT | TGGAGTCGTGTGGTCAGTTG | 223 | 30 |
| *NAA11* | Human | GCTTAAGCATTTCAGGTCCTCAT | GTTGTGCAGTGGCTCTTGTG | 203 | 30 |
| *PP2D1* | Human | CCAGGGTTCAATGGAGTGGC | GACTGCTTGCACATTACCAGTGTTT | 194 | 30 |
| *PPP4R3C* | Human | ACTTGATCCACGCAACATGC | TGGGAGAGTCTACCTTGCCT | 962 | 30 |
| *PRR23A* | Human | GGAATGAACTGCTTCCCACAA | CACTTCCAAAAGGTCCAGTGC | 673 | 30 |
| *PRR23B* | Human | AGCTGGAATTCTGCGCATCT | CAATTGTCCGAACACGCGG | 511 | 30 |
| *PRR23C* | Human | AGACCGTCTCCTGATAGCCG | TGCCTAGGGGTGACTTCCAA | 249 | 30 |
| *PRSS38* | Human | GGTTTGCCTTGCAACTCCAG | TCGTCTGAGGTCTCACCTTG | 96 | 30 |
| *SAXO1* | Human | TGCTTAAGTCTGGACTGCGG | AGGCCCAAAATCTCTCCTTGA | 519 | 30 |
| *SLC22A14* | Human | AAGTGGTGTCAGCAGAGACTG | CCACATACCAAGTCAAACTCATT | 577 | 30 |
| *SPAG11A* | Human | AAGTCATCCTGGAGCACAGC | CTGGCTTGAGACGATCCTGG | 301 | 30 |
| *SPAG11B* | Human | CCCTGCTGTTTCCAGGATCG | TGCTCAGACACTTCCGCAAA | 394 | 30 |
| *SPATA31A1* | Human | GATCATCCAACACTGGGGCA | TCCAAGATGGGATTCCTCGC | 899 | 30 |
| *SPATA31D1* | Human | GAGCCCTGACTCACATTGGTT | TGGGAAACCTTTGAATGTCCC | 193 | 30 |
| *SPATA31D3* | Human | TGTCACCAATGGGTCGAAGG | AGCGACTCCCTTGATGTCTTG | 588 | 30 |
| *SPATA31D4* | Human | TGGAGGTCAGAGGAATCCGT | GACACACCAGGGTGACTTGA | 846 | 30 |
| *SPINT3* | Human | AGCACGAGACACTATCAAGG | CCCTCAGAGCAATCCCGAAT | 251 | 30 |
| *SPINT4* | Human | TGCCATTACAGCCGGAGAAG | AGACCTCAAAGATCCCTGCAA | 125 | 30 |
| *SPINT5P* | Human | CTCTGAAGAAGGGCACCTGTAA | CTCCACAGCCACTGAAGACAA | 96 | 27 |
| *TCP10L2* | Human | GAGACAACGCCCAAATACGC | TACAGGAAGATTCTGCCGCTC | 474 | 30 |
| *TEX13D* | Human | GCTCCAGATCGAGAGGTTTCC | TGATTTGTGGCTCCTGCGAA | 745 | 30 |
| *TEX44* | Human | ATGACCACAGGAGAGCCCTA | GTGAGTGGGACCTGACCTTG | 144 | 30 |
| *TEX48* | Human | GCTGATTCAACAAGGCACCC | AAGCAGCAAATTTTGGGTCG | 200 | 30 |
| *TEX51* | Human | GCTGACTGCAGGACTCACTT | GCCCTGTTCCTTTACTGCCT | 170 | 30 |
| *TLE7* | Human | GCGGGTCTATTTGTCAAGTCC | CAGGAACTTCGTGCTTCCTGAT | 928 | 30 |
| *TPTE2* | Human | AGGAGGCAAAGGAAGAACCG | ATGAACATGTGGCAGGGGTT | 704 | 30 |
| *TRIML1* | Human | GAGGTGTAACCTGGCTGCAT | AAGAGTGGCTCGCTCCTTTC | 809 | 30 |
| *TRPC5OS* | Human | AAACTGCAGGACCATAGTTGGA | ACAGCAAAGCAGACCTGTGATA | 364 | 30 |
| *WFDC10A* | Human | GATGCAGAAAACACAGCTATCCC | GAGGTCCTTGTGCTTCGGAT | 240 | 30 |
| *WFDC10B* | Human | GCATTGTACACAGGAATCAAGGTC | CTCGGATGGAAGGGTTCAGG | 352 | 30 |
| *WFDC13* | Human | GTGGTGTTCTGCCTAGCACT | TGTGTTTGATTCTGTTGCGCTT | 220 | 30 |
| *WFDC8* | Human | AAATTACCGCTGCACACCCT | AGGGAAGAGTGGGCATTGTC | 127 | 30 |
| *WFDC9* | Human | TGCTTAGACAACGAAGAGCCC | GTCATCCTTCAGCCCACAGT | 84 | 30 |
| *1700011L22Rik* | Mouse | CCCAACAACTGTGGCAGGAT | GGCTGCATACAGGGGGACATA | 367 | 30 |
| *1700018B08Rik* | Mouse | GGCTGTATCCAGGAAGGGTT | AACAGACTCGTGCGGACATT | 821 | 30 |
| *1700042G07Rik* | Mouse | TTCTCAGCTTGTTCGAGCCC | CTGAGAACTCGAGGTGACAGAATC | 228 | 30 |
| *4930558K02Rik* | Mouse | TTGGTGCCTAACCAAGAGGT | TGGCTGTACCGTTTTCACTT | 388 | 35 |
| *4932429P05Rik* | Mouse | GAGGTTTGTTAAAATTGCCCCA | GGTGATCCAAAGCAGGGTCA | 829 | 30 |
| *4933412E24Rik* | Mouse | ACTACCCGGGGACTTACCTC | ACTTCTCACTGACAGCCTGC | 772 | 30 |
| *4933424G06Rik* | Mouse | GCAAGAACAACTGCTGAGCC | ATCCGGGTAGCGCAAAAGAA | 495 | 30 |
| *Adam20* | Mouse | TCACTTCACTTGCTATCCTGCT | CTGTGGGTCCACCATTCCTC | 733 | 35 |
| *Bsph1* | Mouse | AATCCAGAGACAGAAGATGGTGC | CATCCTCTGTGCAATCCCAGT | 214 | 30 |
| *Ces5a* | Mouse | GAGCCATCATCGAGGGGTCTG | CTTATGGCTCCCGCTGAGTT | 634 | 30 |
| *Cpa5* | Mouse | TGCTGGGTGCTTTCAGTGAC | CGAAGAACCTGGTCTCCTGT | 521 | 30 |
| *Efcab5* | Mouse | TGCTGATGCAGGTGGAGAAG | ATCCAAGGCCATCACCTTCC | 530 | 30 |
| *Erich6* | Mouse | TCTCTTGACCTATCCGTCTGG | GGCTGATTGTTGCCTGTTGG | 378 | 30 |
| *Etd* | Mouse | CAGAGCCAAGGCTTTCTCCATT | CAGCCACTGTATTCTGGAGGG | 257 | 30 |
| *Fer1l5* | Mouse | GCCTAAACGGTATGAGCTGC | GCTCATGGCCAAATAGTGCG | 298 | 30 |
| *Glt6d1* | Mouse | TTGCTGTTGGCAGTCTCACT | ACAGCACTGGTGCGTCATAA | 697 | 35 |
| *Gm35060* | Mouse | GCTGGCATTGGTGGATACTTTT | GCCCTAGTTGCTGGTCGTT | 106 | 30 |
| *Gm4969* | Mouse | CCCTTTTCCAACGCTCCTCT | TGCCTGCAAGAACTGACACA | 909 | 30 |
| *Gm5294* | Mouse | GCGTACAGCTACATTGCTCTCA | AGCCCAAAACTGAAAGCTCATC | 546 | 30 |
| *Gm5767* | Mouse | CACCAAGAGAGAACCCTGACC | GGAAGCACCCAAACACAAAGAG | 279 | 30 |
| *Gm6657* | Mouse | AGTCATGGAGCTTGCTACGG | TCCTGGGATTCTCGTATCATGT | 481 | 30 |
| *Iqca1l* | Mouse | AGTTCATTGGCATGCTCCCT | TGCTTGTTGTCATACCGGCT | 547 | 30 |
| *Lcn6* | Mouse | ACAGTCGGACAGAGATGGCT | TGGCACTCACTGGAGGATCT | 220 | 30 |
| *Magea4* | Mouse | CTGGGGCTCACCTATGATGG | GTGTAAGCTCTGGGATCGGT | 362 | 30 |
| *Mageb3* | Mouse | GCCCATGCTGAAACAACCAA | TAACAGGCCAAGAATGGATGC | 288 | 30 |
| *Mroh2b* | Mouse | TGATTGGTTCCATGCTGGACT | GCCTTGTAGTCTGTCCACCTC | 572 | 30 |
| *Naa11* | Mouse | TAGAGCATTTCCTGAGCATCGC | GGTTGGGTTGAGATCCCGAG | 847 | 30 |
| *Pet2* | Mouse | TTCCTGTGACGACCTTGTGG | AGGAAAAATGAGAGACGGCAAT | 267 | 30 |
| *Pp2d1* | Mouse | TCCATCAATCAGAAGGCCCAC | TCCAAAACACTCTTAAAGGAGAAGA | 268 | 30 |
| *Prr23a3* | Mouse | TACCCCTAGTTGCAAGGCAC | CATATCCCAGCGCATGCTGA | 417 | 30 |
| *Prr23a3* | Mouse | TACCCCTAGTTGCAAGGCAC | CATATCCCAGCGCATGCTGA | 417 | 30 |
| *Prr23a3* | Mouse | TACCCCTAGTTGCAAGGCAC | CATATCCCAGCGCATGCTGA | 417 | 30 |
| *Prss38* | Mouse | TAGTCGCCGACACCCAAAAA | GTTTCTTCCCCCTGTCAAAGC | 224 | 30 |
| *Saxo1* | Mouse | TGCTAAGTGCTGCCTGTTTG | GCCCAAAATCTCTCCTGCATGT | 425 | 30 |
| *Slc22a14* | Mouse | TTGGGTCTGGACACCAGTCA | GATAGCGGCCCAACTTGTCA | 744 | 35 |
| *Spag11b* | Mouse | CCAAGGAAACTCAGGGGACATC | GTCTCCACCCTTCAGTTGCT | 225 | 35 |
| *Spag11b* | Mouse | CCAAGGAAACTCAGGGGACATC | GTCTCCACCCTTCAGTTGCT | 225 | 35 |
| *Spata31* | Mouse | TTCACCAGGAAGCCTCAGATAC | AAAGGGTGACAGGGTAGTGC | 234 | 30 |
| *Spata31d1b* | Mouse | GGGGACATGGCAGTTTCTCA | AATGGAAACAAACCGCGCC | 862 | 30 |
| *Spata31d1b* | Mouse | GGGGACATGGCAGTTTCTCA | AATGGAAACAAACCGCGCC | 862 | 30 |
| *Spata31d1b* | Mouse | GGGGACATGGCAGTTTCTCA | AATGGAAACAAACCGCGCC | 862 | 30 |
| *Spint3* | Mouse | CGCAGAACCAAATAATGTGCC | CCTCCATAGCGGAACCACTC | 141 | 30 |
| *Spint4* | Mouse | AGACTACAACGATCCCTGTCTG | CGTGCTCTCTGTAGCAAGGT | 333 | 30 |
| *Spint5* | Mouse | TGAGTCTCTCCTGTCTGCTGT | TCTTTTCGCTCCAAGGTGCC | 169 | 30 |
| *Tcp10c* | Mouse | CACTGGCTGACATCTGGTCC | CCTCTGCTCTAGGGCCAAAC | 925 | 30 |
| *Tex13c1* | Mouse | TTTCAGCAGACGACATGCCT | CCATTCTAACTCCACCATCTTCCT | 445 | 30 |
| *Tex44* | Mouse | AGCAAAGCTAGATCGCCAGG | ATGGAGCGCATGTAGTCGTT | 197 | 30 |
| *Tex48* | Mouse | TGGGGCTCAAAGGAAACAGAC | CTTTAATTGCAGGCAGCCCC | 645 | 30 |
| *Tle7* | Mouse | TGGCTCAAGATTCCAACCAGG | GTGAGGACACGATTCCGAGG | 327 | 30 |
| *Tpte* | Mouse | AGCACTGGCTACGACACAAA | CAGTCACACCGATGACCACA | 706 | 30 |
| *Triml1* | Mouse | GCTGCGAAAATTCAGCACAGA | GTAATTGCTGCCTGGTCCCT | 113 | 30 |
| *Trpc5os* | Mouse | ATCTCGCGGATCTTTCCGAC | TCCTAATAGTACAGGTAAAGCAAGA | 365 | 30 |
| *Wfdc10* | Mouse | TCTACCCAGAGAGTGGGTCC | GAAGGAGGCAGGTGCTTGTT | 176 | 35 |
| *Wfdc13* | Mouse | ACACATCGAGCCAGCATCAT | AGGTTCCAGGATGTACTTCAGA | 123 | 35 |
| *Wfdc8* | Mouse | TGTGTGTCCTATCTGCTGGTTC | GACTGCGCTTCATGGGTGAG | 702 | 37 |
| *Wfdc9* | Mouse | AGACTACAAACCACAGCAGCA | CAAGGTCATCCTTGAGGCTTCC | 149 | 35 |
| *Wfdc9* | Mouse | AGACTACAAACCACAGCAGCA | CAAGGTCATCCTTGAGGCTTCC | 149 | 35 |
