# Supplementary figures and images for: Large-scale discovery of male reproductive tract-specific genes through analysis of RNA-seq datasets
Source: BMC Biol. 2020 Aug 19;18:103. doi: 10.1186/s12915-020-00826-z (PMC7436996; doi:10.1186/s12915-020-00826-z)

A

Human (720 Genes)

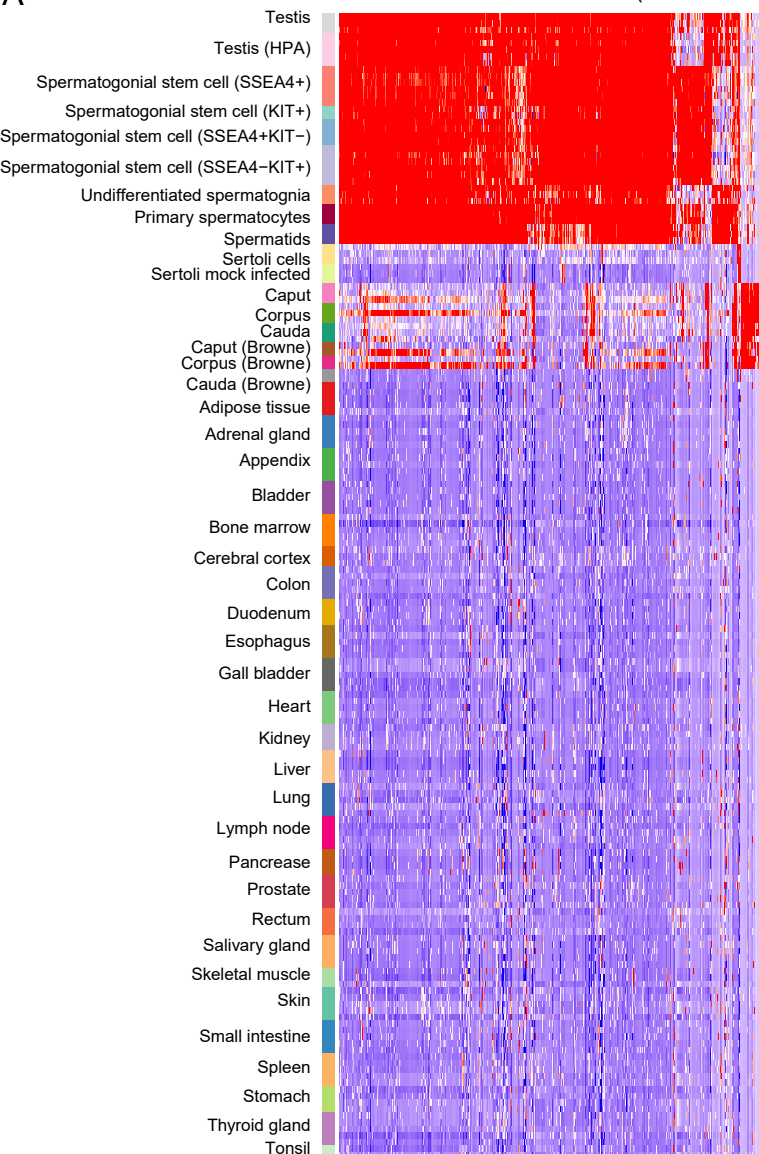

-1 -0.5 0 0.5 1  
z-score(log2CPM)

B

Mouse(1062 Genes)

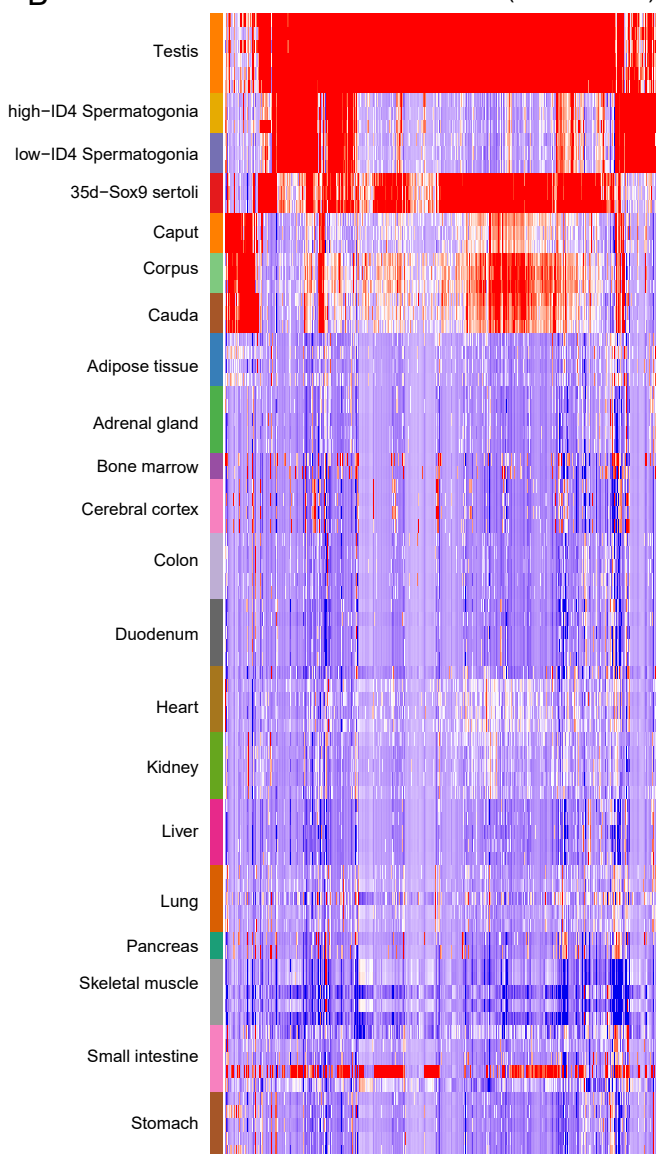

-1 -0.5 0 0.5 1  
z-score(log2CPM)

Supplement: Supplementary file 2 — Additional file 2: Fig. S1. Genes that passed the TPM and FDR filters in at least one of the measured reproductive tissues or cells were visualized using a heatmap of the RUVr batch corrected log2 CPM gene expression values for the human (A) and mouse (B) samples. [file 12915_2020_826_MOESM2_ESM.pdf]

A

HUMAN

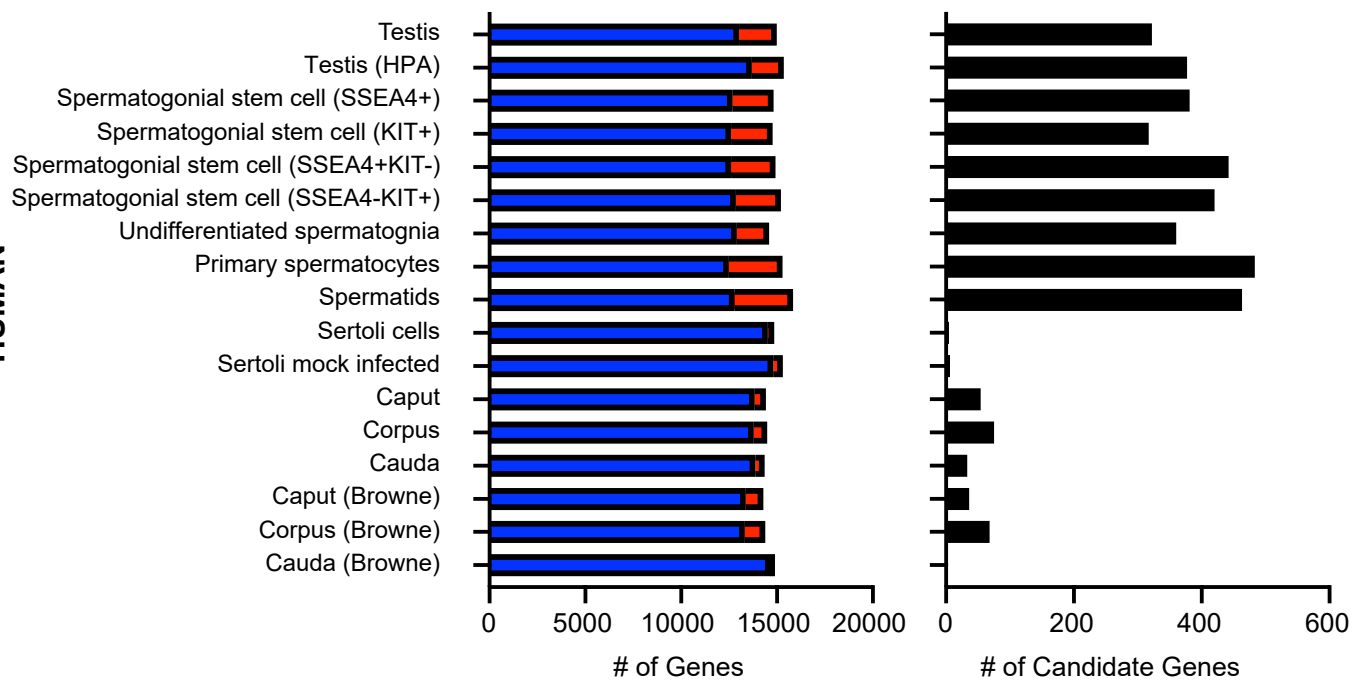

B

MOUSE

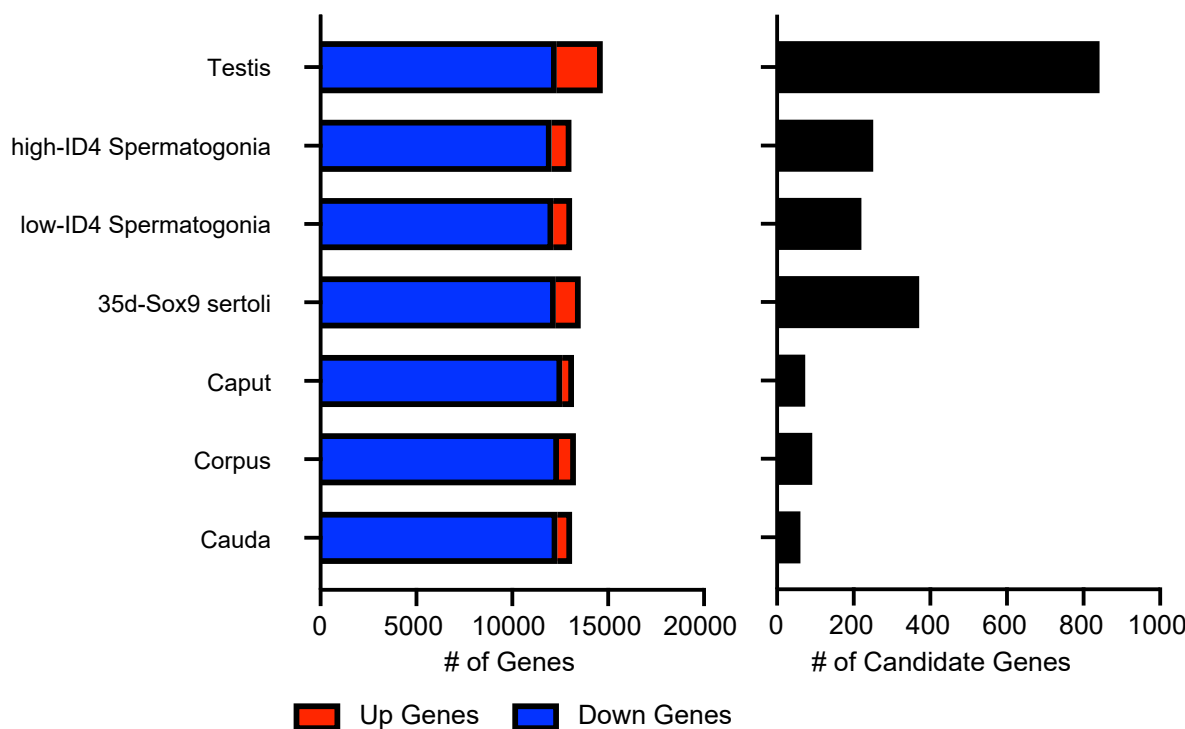

Supplement: Supplementary file 7 — Additional file 7: Fig. S2. Summary of number of statistically significant up and down-regulated genes, and quantification of candidate genes with respect to the individual reproductive tissue or cell of interest. The plots in panels (A) and (B) summarizes the number of statistically significant human or mouse genes respectively, that are up-regulated or down-regulated in each reproductive tissue or cell of interest compared to the non-reproductive tissue with maximal gene expression. Red columns depict the number genes that are up-regulated and blue columns depict the number genes that are down-regulated. Changes in gene expression were considered statistically significant for an FDR of less than or equal to 0.05. The total number of candidate genes are designated by the black columns. Candidate genes are genes that passed the FDR and TPM expression value filters. [file 12915_2020_826_MOESM7_ESM.pdf]

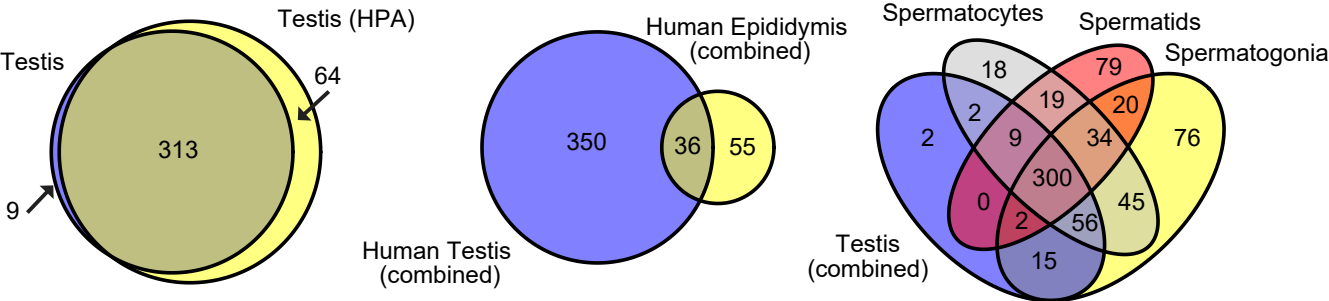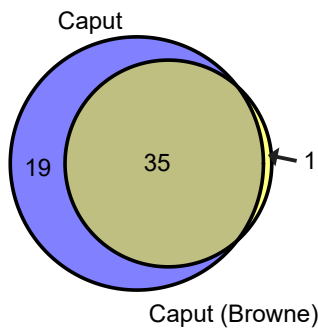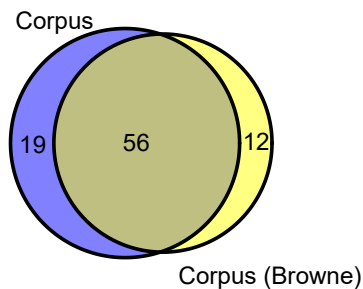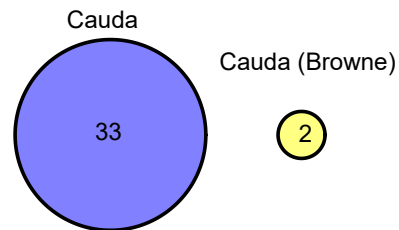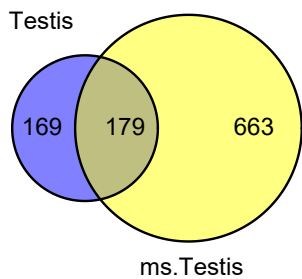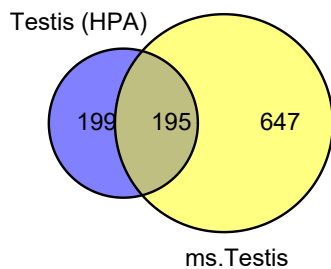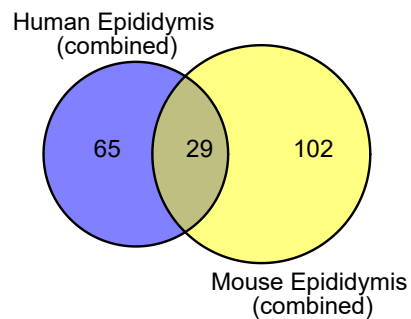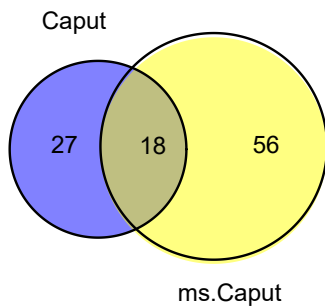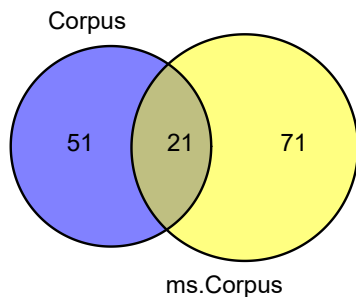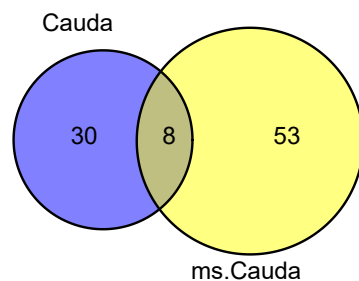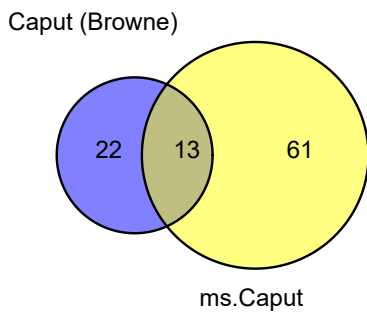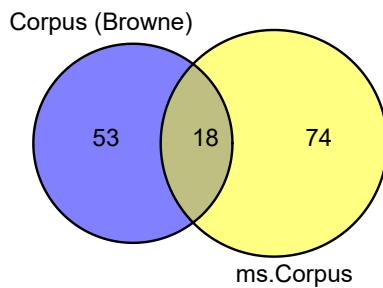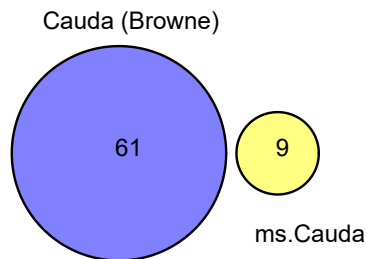

Supplement: Supplementary file 8 — Additional file 8: Fig. S3. Venn diagrams comparing the overlap between the candidate male reproductive genes identified by the indicated reproductive tissues. The human testis combined gene list is the list of genes from both new samples we isolated and from previously published testis samples. The human epididymis combined gene list is the list of genes identified in either previously published samples or the newly generated samples across all sections of the epididymis. Lastly, the mouse epididymis combined gene list is the list combined list of genes identified across all three sections of the mouse epididymis. [file 12915_2020_826_MOESM8_ESM.pdf]

A

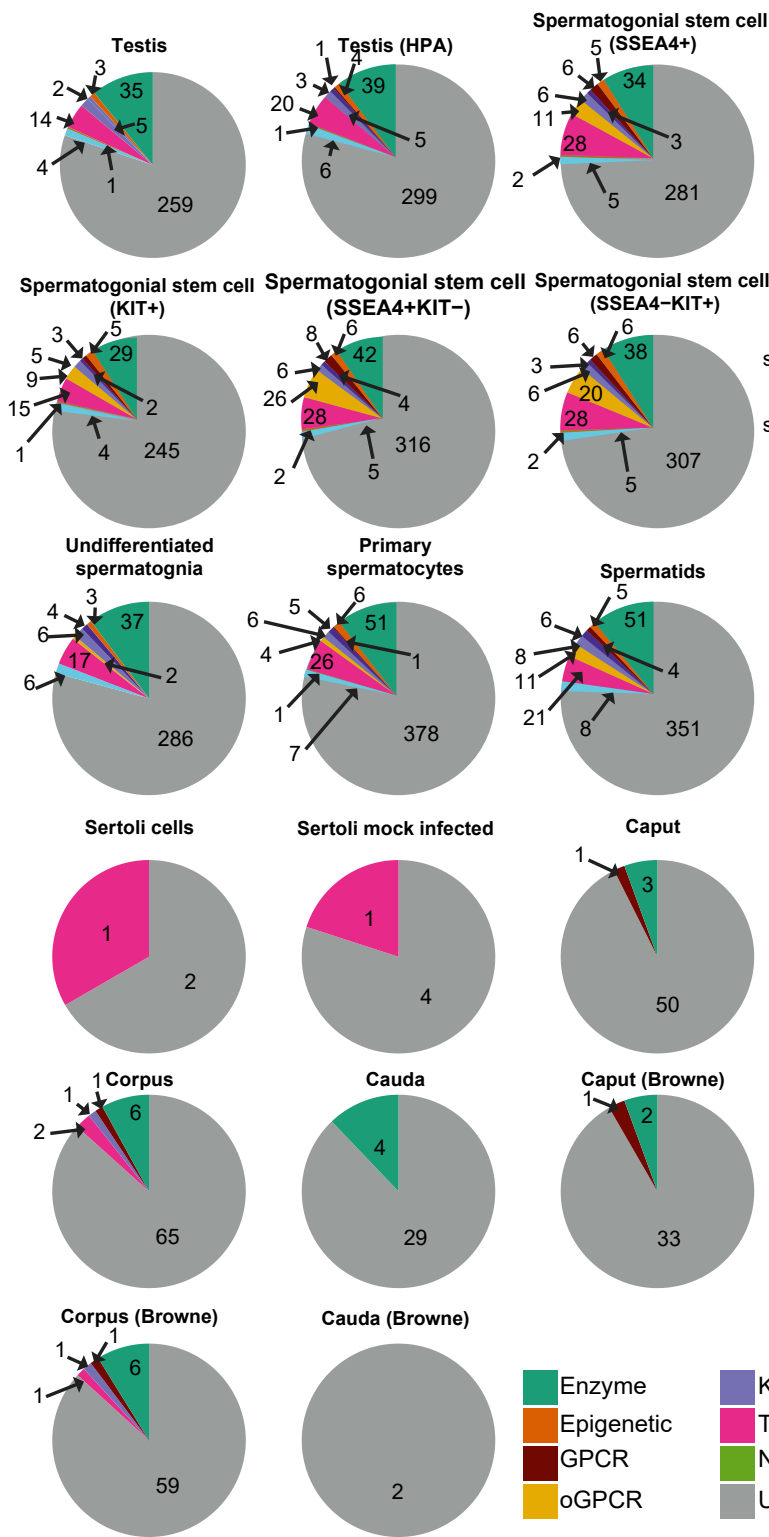

B

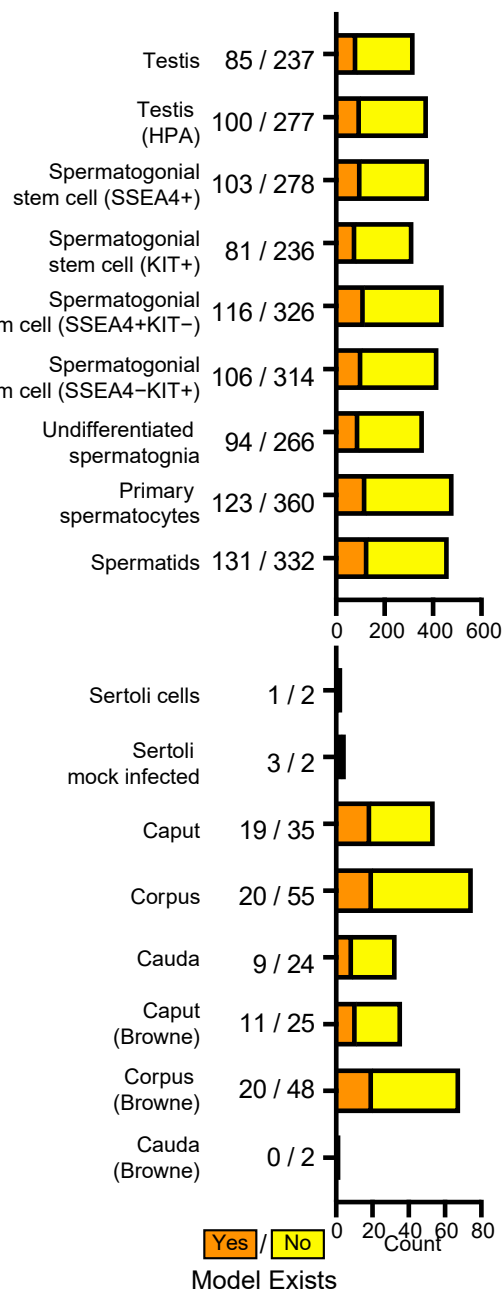

Supplement: Supplementary file 10 — Additional file 10: Fig. S4. Classification of genes into different protein families and identification of the existence of an experimental mouse model. Each candidate human gene was classified as an enzyme (enzyme), chromosome and histone modifiers (epigenetic), G-protein-coupled receptor (GPCR), orphan G-protein-couple receptor (oGPCR), kinase (kinase), transcription factor (TF), nuclear receptor (NR), ion channel (IC), chromosome and histone modifying transcript factor (TF; epigenetic), transporter (transporter) and unknown (A). The total number of candidate genes identified in our search for mouse models were plotted. Orange columns designate the number of candidate genes where a model was identified while yellow designates candidate genes where a model was not identified (B). [file 12915_2020_826_MOESM10_ESM.pdf]

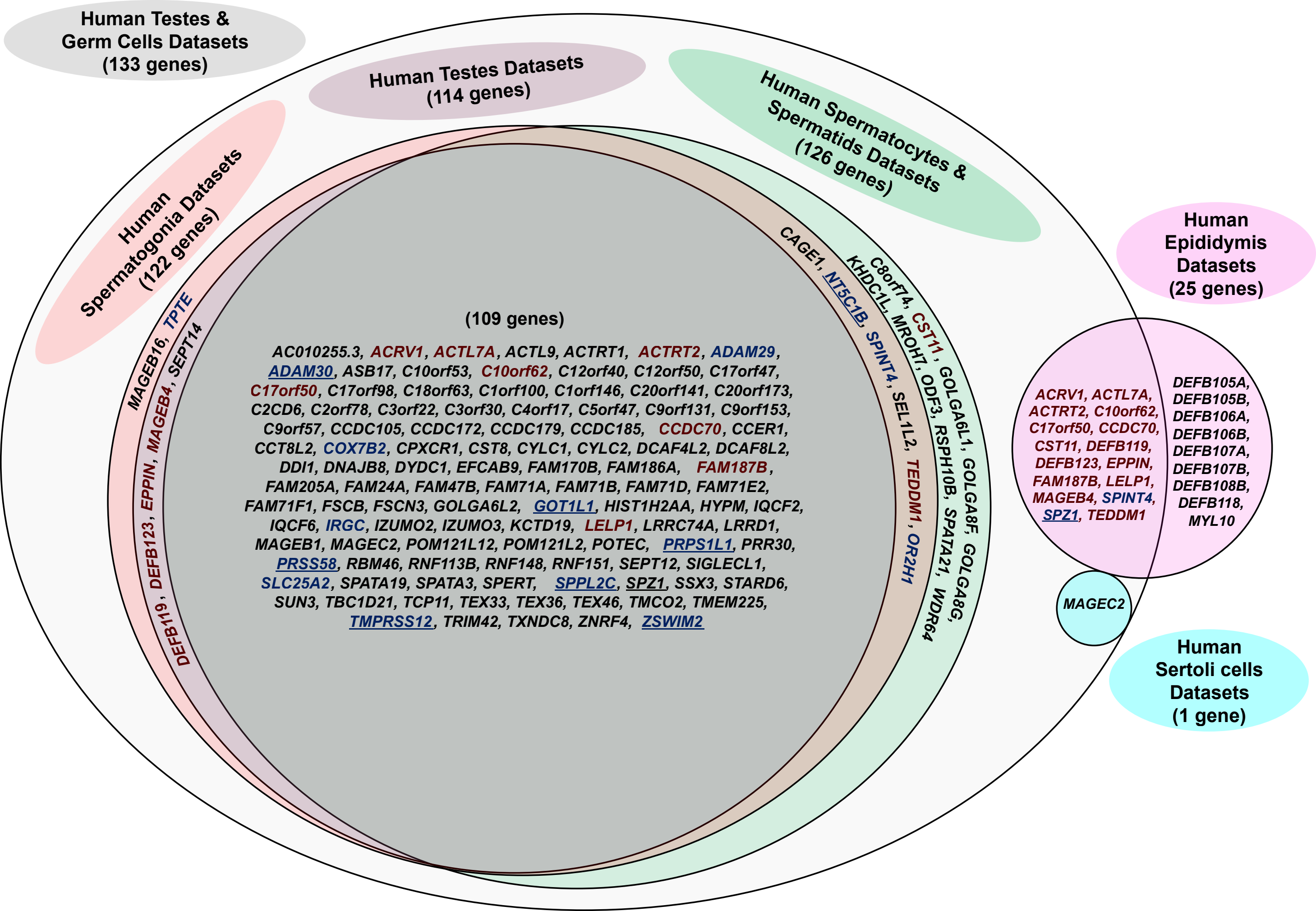

Supplement: Supplementary file 13 — Additional file 13: Fig. S5. One-hundred and forty-two previously identified human male reproductive tract-specific genes that remain without a reported mouse model. The listed genes were identified in one or more datasets as indicated in the Venn diagram. Underlined genes were also identified in our studies as reproductive tract-specific in mouse. Genes written in blue encode either enzymes, kinases, GPCRs, oGPCRs, transporters, transcription factors, or proteins involved in epigenetic regulation. Genes written in dark red were identified in both testis (testis and/or testis cell) and in epididymis. [file 12915_2020_826_MOESM13_ESM.pdf]

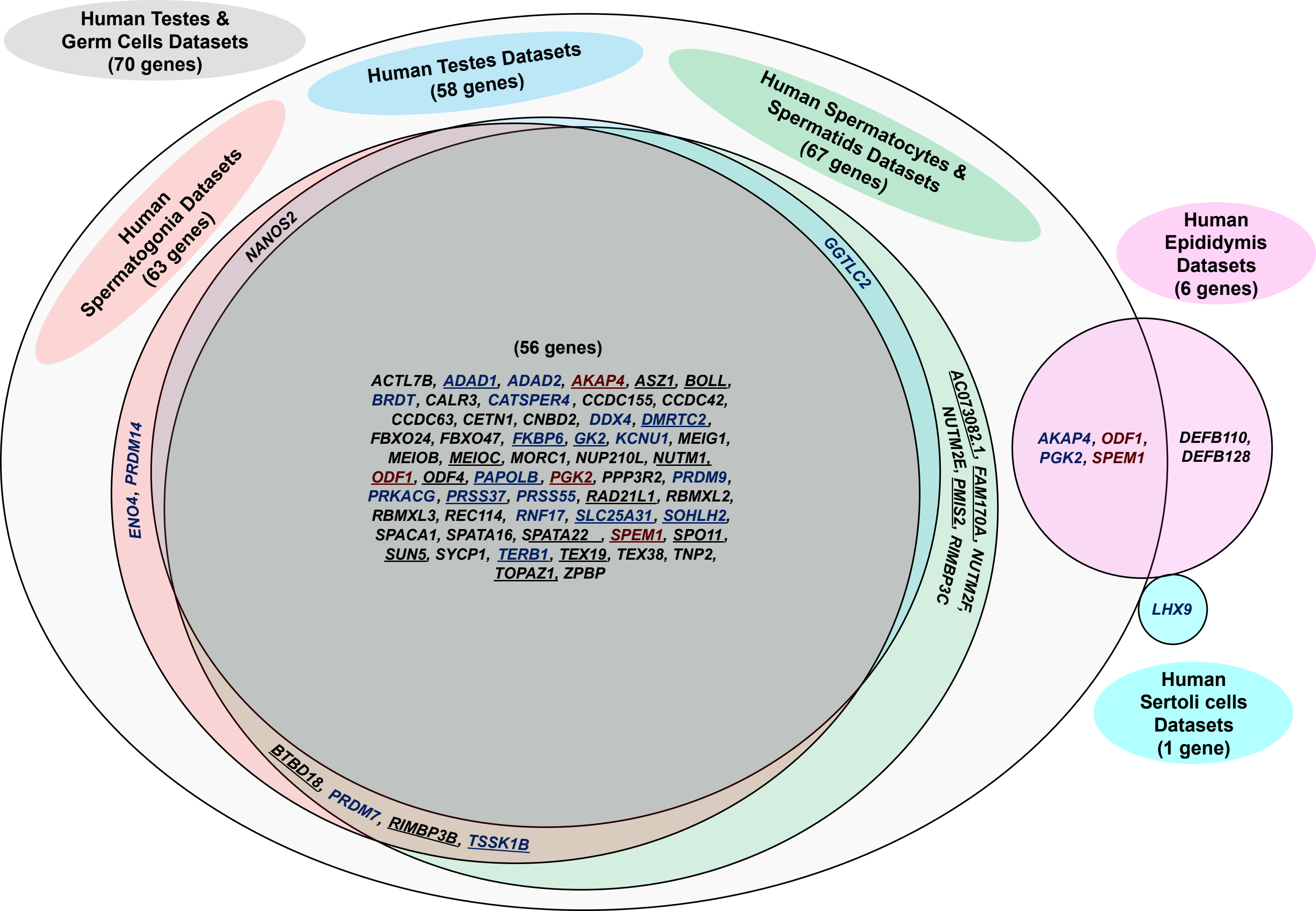

Supplement: Supplementary file 14 — Additional file 14: Fig. S6. Seventy-three human male reproductive tract-specific genes that each have a reported mouse model with male infertility phenotype. The listed genes were identified in one or more datasets as indicated in the Venn diagram. Underlined genes were also identified in our studies as reproductive tract-specific in mouse. Genes written in blue encode either enzymes, kinases, GPCRs, oGPCRs, transporters, transcription factors, or proteins involved in epigenetic regulation. Genes written in dark red were identified in both testis (testis and/or testis cell) and in epididymis. [file 12915_2020_826_MOESM14_ESM.pdf]

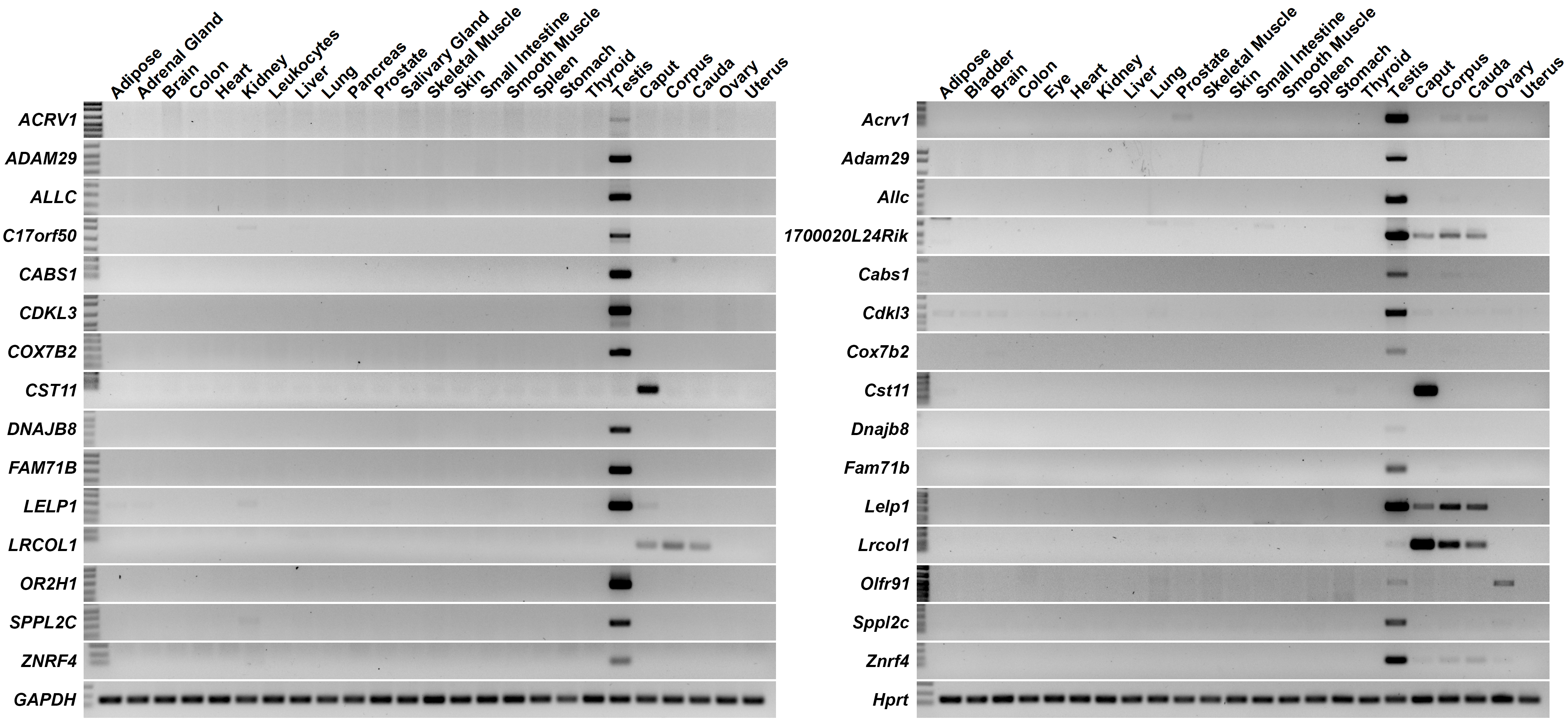

Supplement: Supplementary file 15 — Additional file 15: Fig. S7. RT-PCR confirmation of reproductive tract-specificity in both humans (A) and mice (B). The genes listed in this figure were identified through our studies and previous studies, but currently remain without a reported mouse model. GAPDH and Hprt are included as housekeeping genes. [file 12915_2020_826_MOESM15_ESM.tif]

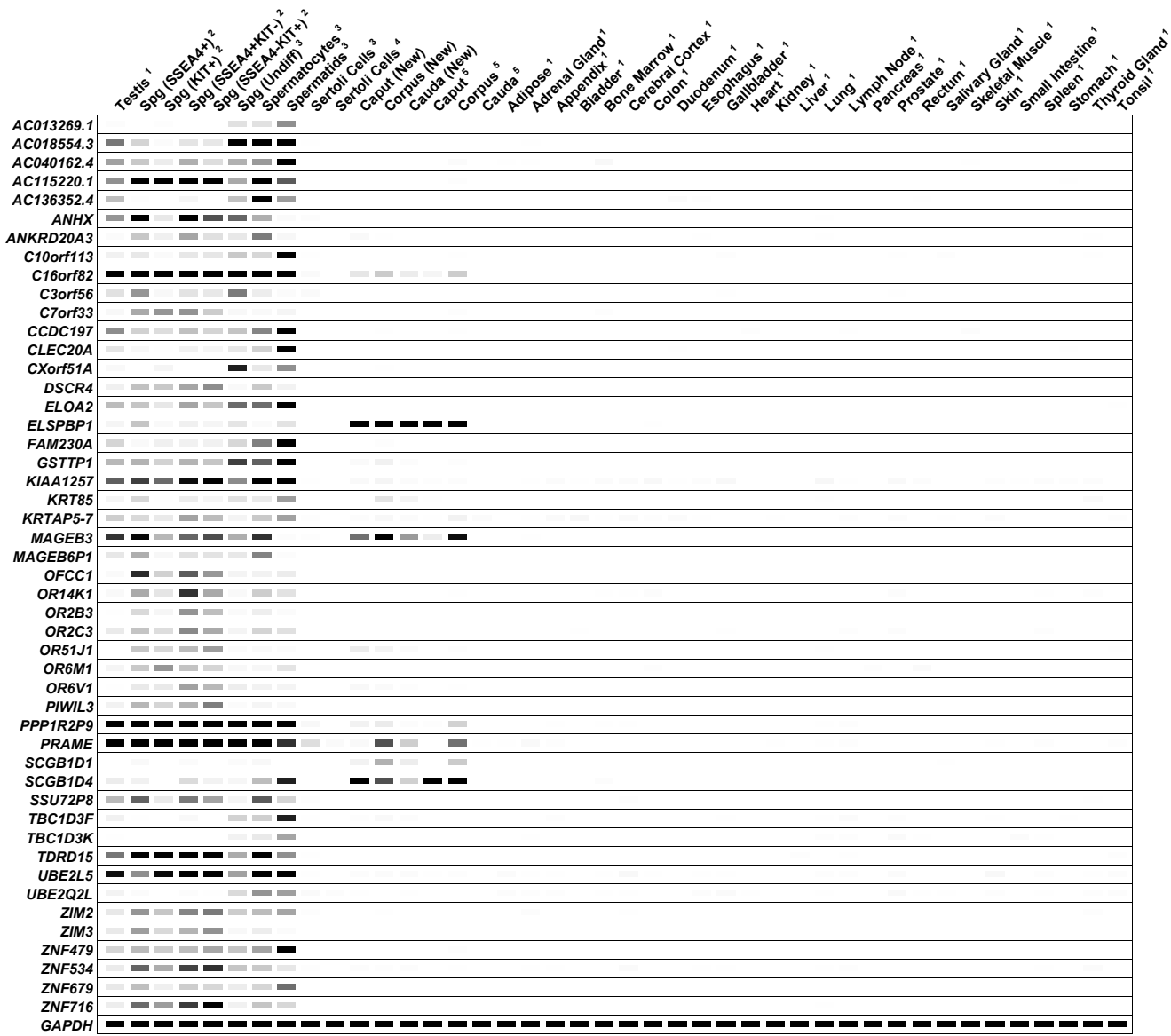

Supplement: Supplementary file 17 — Additional file 17: Fig. S9. Novel reproductive tract-specific human genes that do not have any equivalent mouse orthologs. These genes may serve as potential contraceptive targets, however functional validation would need to be carried out in another model organism than mouse, such as rat or marmoset, which do have orthologs to these genes. The digital PCR (heatmap) depicts the average transcripts per million (TPM) value per tissue per gene from the indicated human RNA-seq datasets as processed in parallel through our bioinformatics pipeline. White = 0 TPM, Black ≥30 TPM. The expression profile of the human housekeeping gene, GAPDH, is included as reference. For data obtained from published datasets, superscript values reference the dataset publication as previously mentioned. [file 12915_2020_826_MOESM17_ESM.pdf]

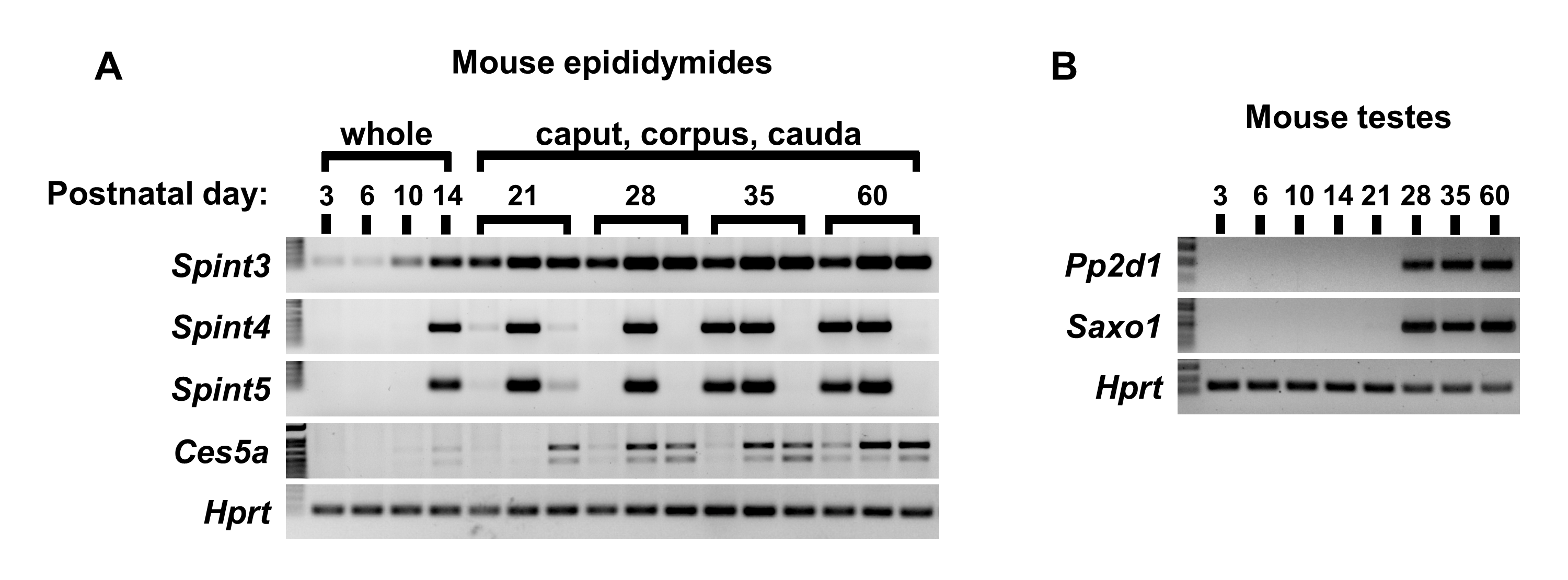

Supplement: Supplementary file 19 — Additional file 19: Fig. S10. Developmental expression pattern of Spint3, Spint4, Spint5, Pp2d1, and Saxo1 in epididymis and testis of postnatal and adult mice. Whole epididymides were used at postnatal days 3, 6, 10, and 14 and epididymis segments (caput, corpus, and cauda) were used at postnatal days 21, 28, 35, and 60. Whole testes were used at all time points. The housekeeping gene, Hprt, was used as reference. [file 12915_2020_826_MOESM19_ESM.tif]

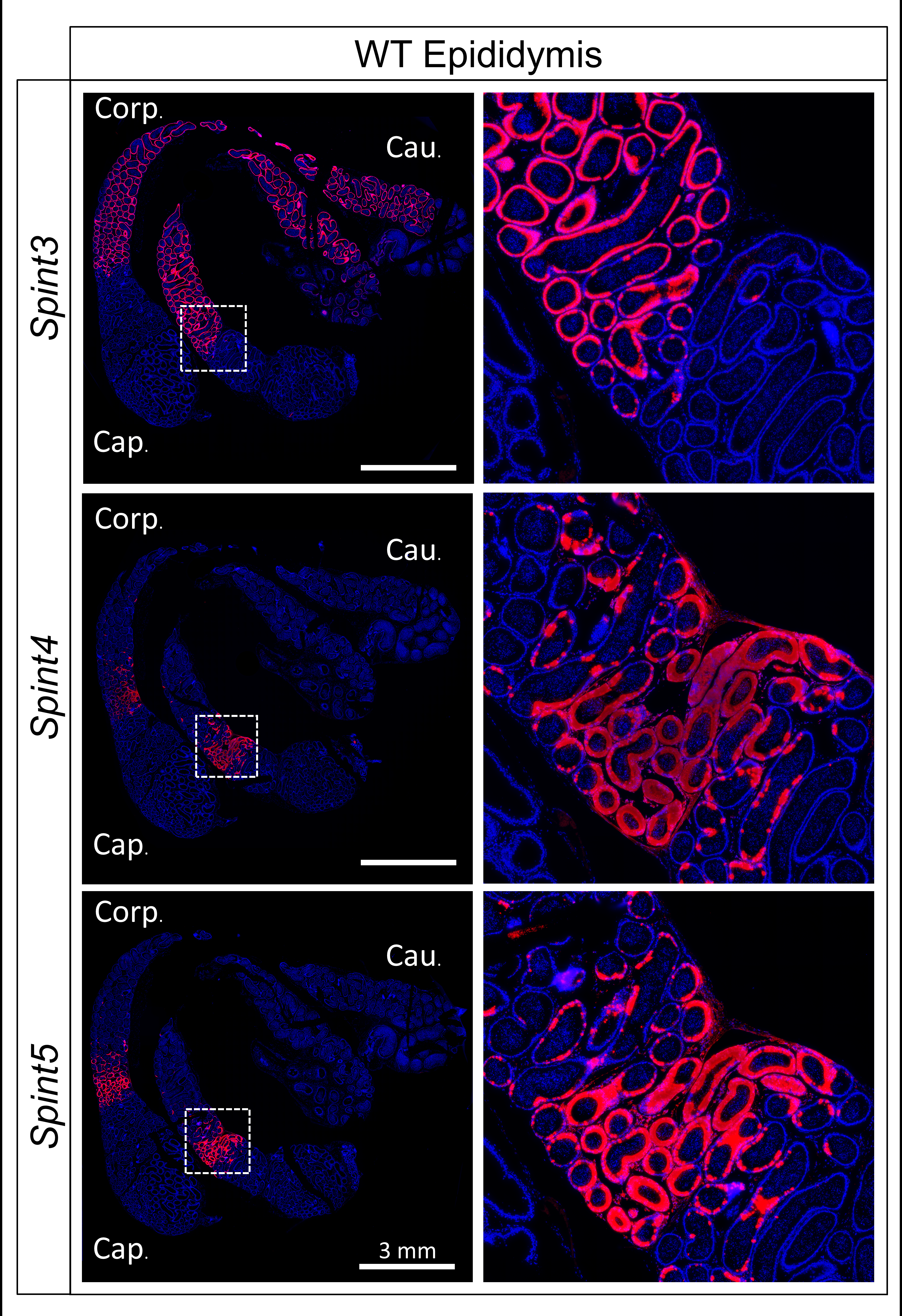

Supplement: Supplementary file 20 — Additional file 20: Fig. S11. Multi-channel fluorescence images of bilateral epididymis serial sections stained with custom RNAscope probes targeting either Spint3, Spint4, or Spint5 mRNA (red) and DAPI (blue). The position of Caput (Cap), Corpus (Cor), and Cauda (Cau) is labeled in the overview image (left column). The position of the magnification over the epididymis is the same for all three sections (right column). [file 12915_2020_826_MOESM20_ESM.tif]

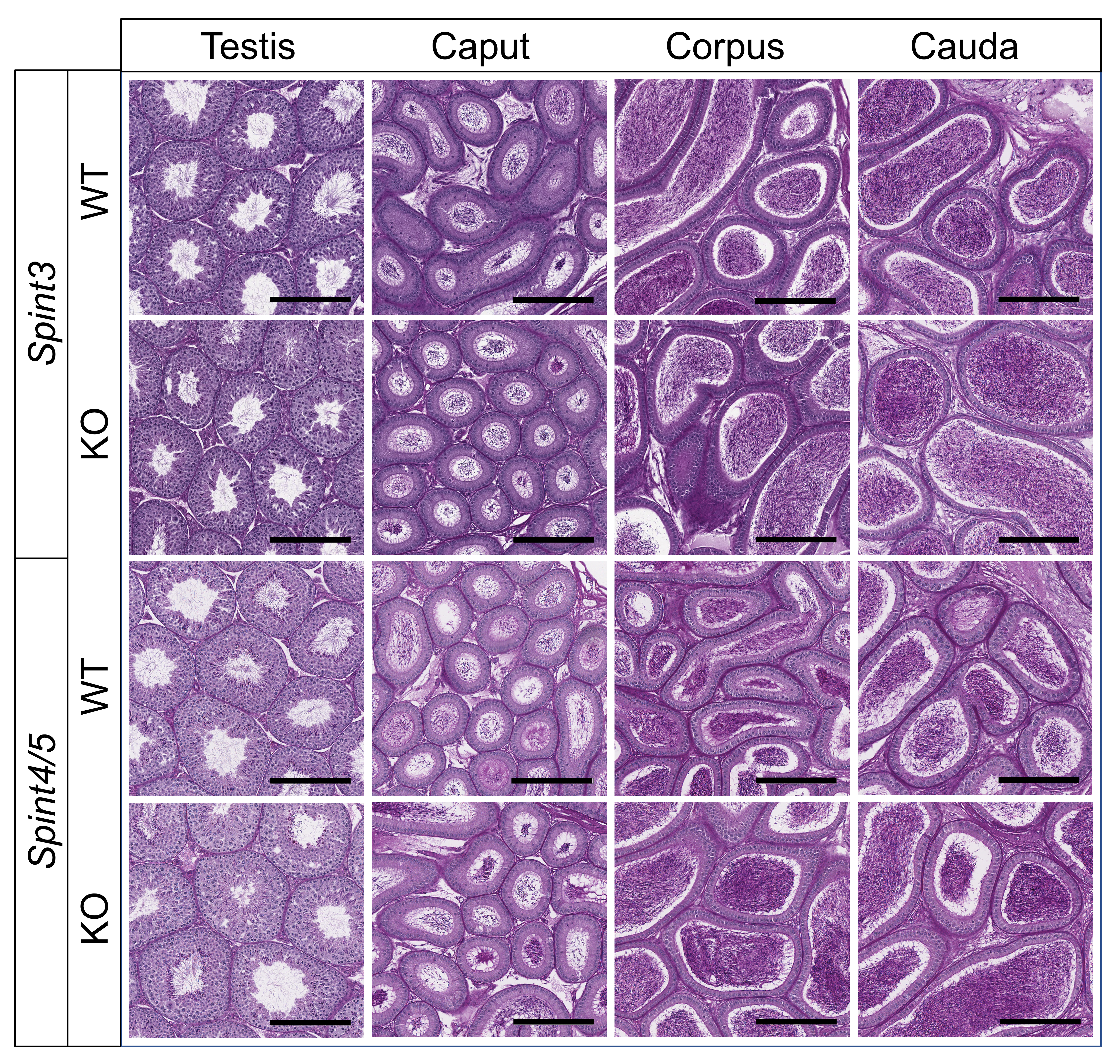

Supplement: Supplementary file 21 — Additional file 21: Fig. S12. Representative periodic acid-Schiff staining of Spint3 and Spint4/5 knockout and littermate control (wild-type) testes and epididymis segments (caput, corpus, and cauda) at 3 months of age. [file 12915_2020_826_MOESM21_ESM.tif]

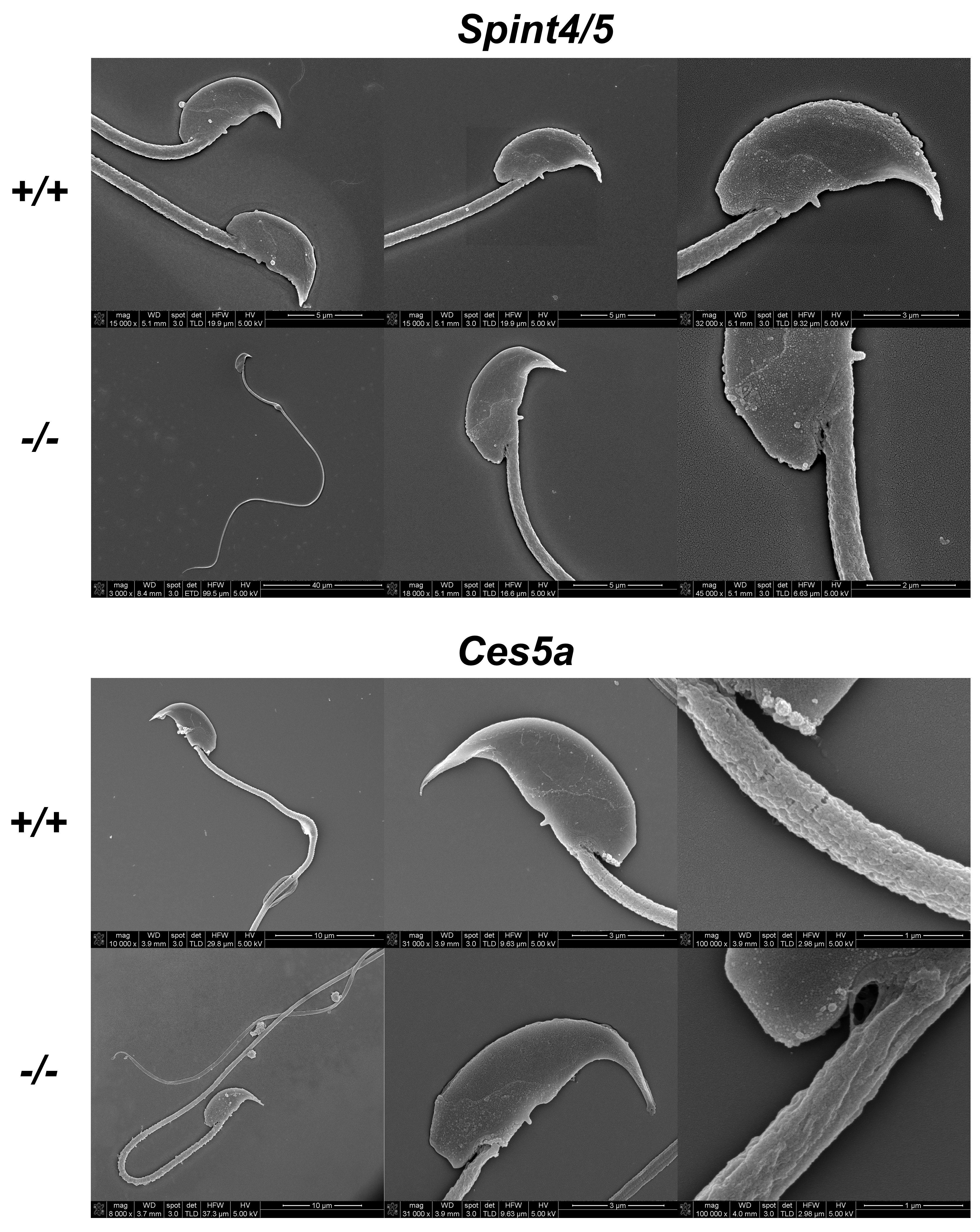

Supplement: Supplementary file 23 — Additional file 23: Fig. S14. Representative scanning electron microscopy images of Spint4/5 and Ces5a KO and littermate control mouse sperm. [file 12915_2020_826_MOESM23_ESM.tif]

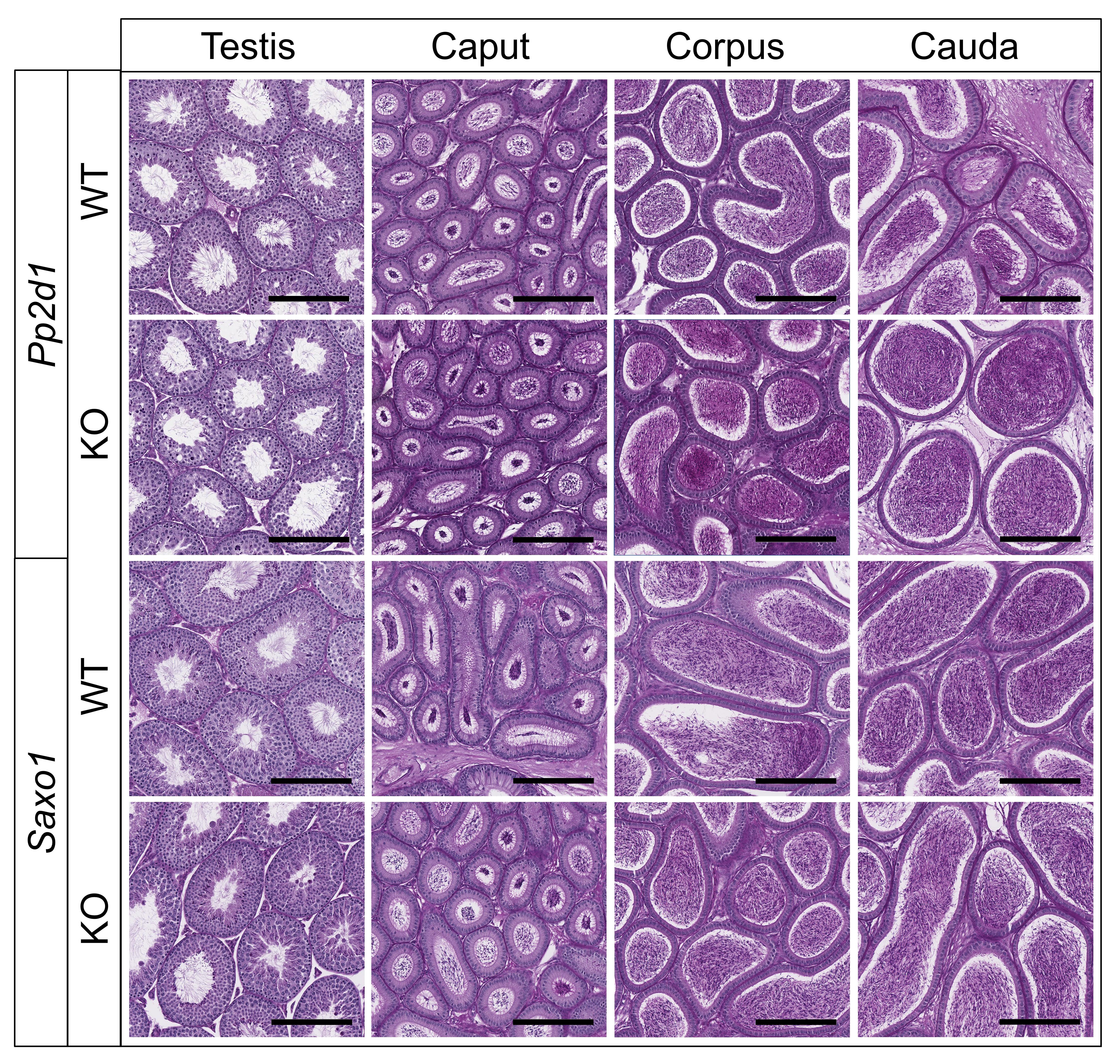

Supplement: Supplementary file 24 — Additional file 24: Fig. S15. Representative periodic acid-Schiff staining of Pp2d1 and Saxo1 knockout and littermate control (wild-type) testes and epididymis segments (caput, corpus, and cauda) at 3 months of age. [file 12915_2020_826_MOESM24_ESM.tif]
