# Supplementary material for: Large-scale discovery of male reproductive tract-specific genes through analysis of RNA-seq datasets
Source: BMC Biol. 2020 Aug 19;18:103. doi: 10.1186/s12915-020-00826-z (PMC7436996; doi:10.1186/s12915-020-00826-z)
Supplement: Supplementary file 16 — Additional file 16: Fig. S8. Eighty-nine novel human genes without a mouse ortholog. The listed genes were identified in one or more datasets as indicated in the Venn diagram. Genes written in blue encode either enzymes, kinases, GPCRs, oGPCRs, transporters, transcription factors, or proteins involved in epigenetic regulation. Genes written in dark red were identified in both testis (testis and/or testis cell) and in epididymis. [file 12915_2020_826_MOESM16_ESM.pdf]

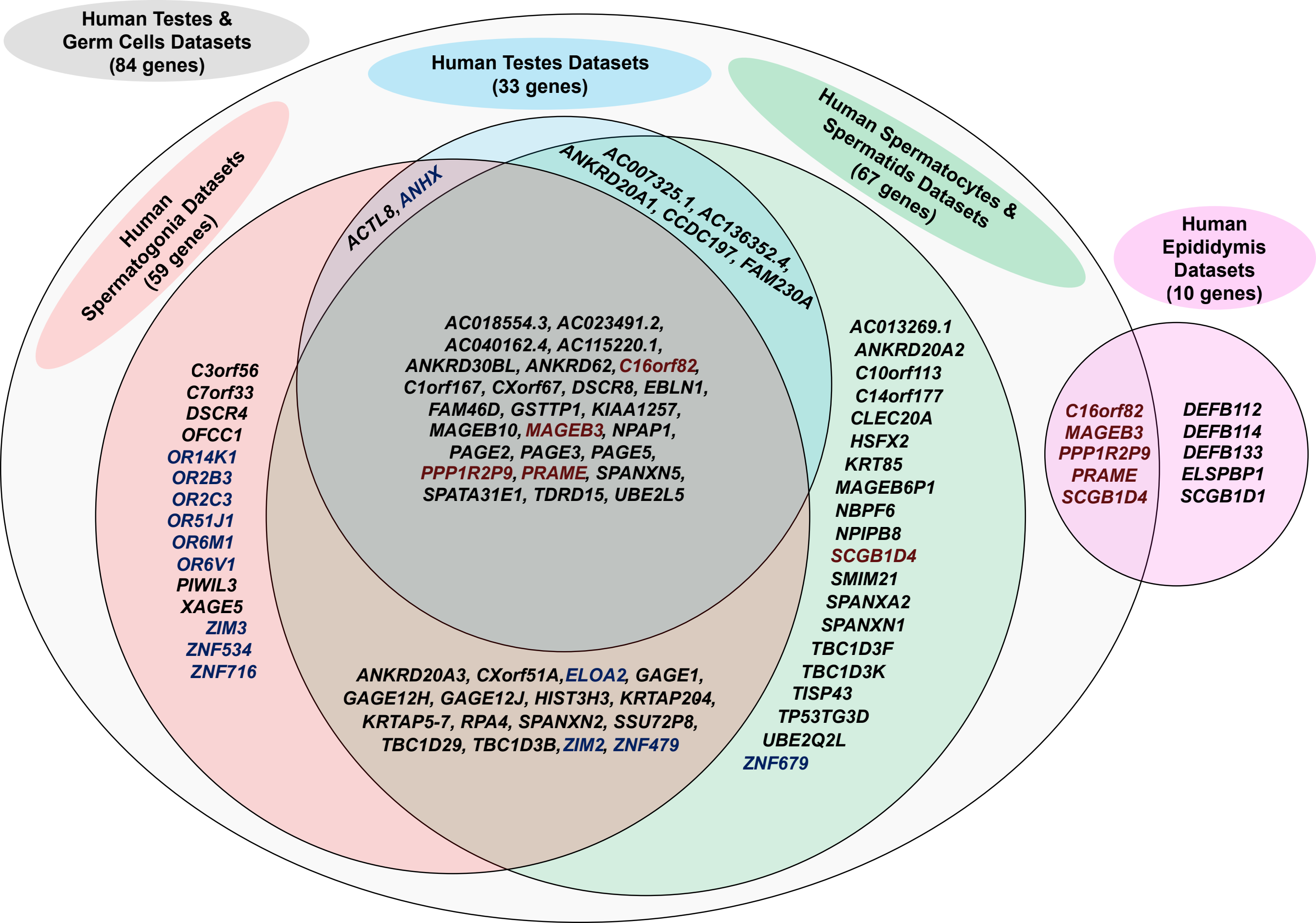

Human Testes & Germ Cells Datasets (84 genes)
Human Testes Datasets (33 genes)
Human Spermatocytes & Spermatids Datasets (67 genes)
Human Spermatogonia Datasets (59 genes)
Human Epididymis Datasets (10 genes)
ACTL8, ANHX
AC007325.1, AC136352.4, ANKRD20A1, CCDC197, FAM230A
AC018554.3, AC023491.2, AC040162.4, AC115220.1, ANKRD30BL, ANKRD62, C16orf82, C1orf167, CXorf67, DSCR8, EBLN1, FAM46D, GSTTP1, KIAA1257, MAGEB10, MAGEB3, NPAP1, PAGE2, PAGE3, PAGE5, PPP1R2P9, PRAME, SPANXN5, SPATA31E1, TDRD15, UBE2L5
C3orf56
C7orf33
DSCR4
OFCC1
OR14K1
OR2B3
OR2C3
OR51J1
OR6M1
OR6V1
PIWIL3
XAGE5
ZIM3
ZNF534
ZNF716
ANKRD20A3, CXorf51A, ELOA2, GAGE1, GAGE12H, GAGE12J, HIST3H3, KRTAP204, KRTAP5-7, RPA4, SPANXN2, SSU72P8, TBC1D29, TBC1D3B, ZIM2, ZNF479
AC013269.1
ANKRD20A2
C10orf113
C14orf177
CLEC20A
HSFX2
KRT85
MAGEB6P1
NBPF6
NPIPB8
SCGB1D4
SMIM21
SPANXA2
SPANXN1
TBC1D3F
TBC1D3K
TISP43
TP53TG3D
UBE2Q2L
ZNF679
C16orf82
MAGEB3
PPP1R2P9
PRAME
SCGB1D4
DEFB112
DEFB114
DEFB133
ELSPBP1
SCGB1D1
